# NNMT activation can contribute to the development of fatty liver disease by modulating the NAD^**+**^ metabolism

**DOI:** 10.1038/s41598-018-26882-8

**Published:** 2018-06-05

**Authors:** Motoaki Komatsu, Takeshi Kanda, Hidenori Urai, Arata Kurokochi, Rina Kitahama, Shuhei Shigaki, Takashi Ono, Hideo Yukioka, Kazuhiro Hasegawa, Hirobumi Tokuyama, Hiroshi Kawabe, Shu Wakino, Hiroshi Itoh

**Affiliations:** 1 Department of Internal Medicine, School of Medicine, Keio University, Shinjuku-ku, Tokyo, Japan; 2 Shionogi & Company, Limited, Osaka, Japan; 3 Health Centre, Keio University, Yokohama, Japan

## Abstract

Nicotinamide N-methyltransferase (NNMT) catalyses the reaction between nicotinamide (NAM) and *S*-adenosylmethionine to produce 1-methylnicotinamide and *S*-adenosylhomocysteine. Recently, this enzyme has also been reported to modulate hepatic nutrient metabolism, but its role in the liver has not been fully elucidated. We developed transgenic mice overexpressing NNMT to elucidate its role in hepatic nutrient metabolism. When fed a high fat diet containing NAM, a precursor for nicotinamide adenine dinucleotide (NAD)^+^, these NNMT-overexpressing mice exhibit fatty liver deterioration following increased expression of the genes mediating fatty acid uptake and decreased very low-density lipoprotein secretion. NNMT overactivation decreased the NAD^+^ content in the liver and also decreased gene activity related to fatty acid oxidation by inhibiting NAD^+^–dependent deacetylase Sirt3 function. Moreover, the transgenic mice showed liver fibrosis, with the induction of inflammatory and fibrosis genes. Induced NNMT expression decreased the tissue methylation capacity, thereby reducing methylation of the connective tissue growth factor (CTGF) gene promoter, resulting in increased *CTGF* expression. These data indicate that NNMT links the NAD^+^ and methionine metabolic pathways and promotes liver steatosis and fibrosis. Therefore, targeting NNMT may serve as a therapeutic strategy for treating fatty liver and fibrosis.

## Introduction

Non-alcoholic fatty liver disease (NAFLD), the most common chronic liver disorder, is involved in various metabolic disorders, such as obesity, type-2 diabetes, and atherosclerosis^1^. Both the increased lipid influx into the liver and the de novo lipid synthesis contribute to hepatic lipid accumulation. Decreased lipid utilization, via fatty acid oxidation and lipid export, also leads to hepatic steatosis^2^. Some individuals with NAFLD develop non-alcoholic steatohepatitis (NASH), which is accompanied by liver inflammation and liver fibrosis. Although abnormal hepatic lipid accumulation follows a benign course, NASH may progress to liver cirrhosis and hepatocellular carcinoma. Therefore, additional mechanisms have been suggested to be involved in the progression from NAFLD to NASH.

**Figure 1:**
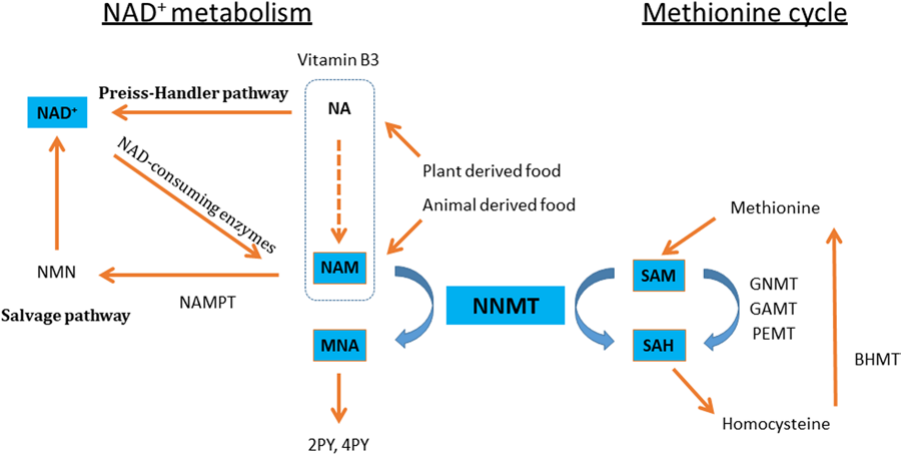
NNMT links NAD^+^ and methionine metabolism. NA is metabolized to NAM via the amidation pathway. NA and NAM are the common forms of Vitamin B_3_, and are converted to NAD^+^ through the Preiss-Handler and salvage pathways, respectively. NAD^+^ is metabolized to NAM by the enzymatic activities of NAD^+^-consuming enzymes (Sirtuins, PARPs, cADPR synthases). NNMT methylates NAM, using SAM as a methyl donor, yielding MNA and SAH. MNA and its oxidation products, 2PY and 4PY are major metabolites of NAM. GNMT, GAMT and PEMT are the main methyltransferases mediating SAM catabolism in the liver. *BHMT1* is the enzyme for the re-methylation of homocysteine to generate methionine. Abbreviations: NA, nicotinic acid; NAM, nicotinamide; NAD, nicotinamide adenine dinucleotide; NMN, nicotinamide mononucleotide; PARP, poly adenosine diphosphate ribose polymerase; cADPR, cyclic adenosine diphosphate-ribose; NAMPT, nicotinamide phosphoribosyltransferase; NNMT, nicotinamide N-methyltransferase; MNA, 1-methylnicotinamide; 2PY, N-methyl-2-pyridone-5-carboxamide; 4PY, N-methyl-4-pyridone-5-carboxamide; SAM, S-adenosylmethionine; SAH, *S*-adenosylhomocysteine; GNMT, glycine N-methyltransferase; GAMT, guanidinoacetate N-methyltransferase; PEMT, phosphatidylethanolamine N-methyltransferase; BHMT, betaine homocysteine methyltransferase.

Nicotinamide adenine dinucleotide (NAD^+^) metabolism is an emerging therapeutic target for metabolic diseases, such as obesity, diabetes and NAFLD^3^. This is because NAD^+^ is an essential cofactor for NAD^+^-dependent Sirtuin deacetylases, improving metabolic derangement in various tissues. Indeed, NAD^+^ supplementation with NAD^+^ precursors ameliorates fatty liver disease^4,5^. Niacin, also known as nicotinic acid (NA) or vitamin B_3_, and its metabolite (nicotinamide [NAM]) are precursors for NAD^+^ biosynthesis (Fig. 1)^3^. Therapy involving niacin supplementation has been used to lower plasma lipid levels^6^; however, its practical use is often limited by side effects, including hepatotoxicity^7^. High doses of vitamin B_3_ can induce hepatic inflammation and fibrosis in humans^8,9^, with NAM reportedly causing hepatotoxicity and triglyceride accumulation in the liver^6,8,10^. These data implied an important role of NAD^+^ metabolism in the pathogenesis of fatty liver as well as liver fibrosis partly through the modulation of sirtuin activity.

Nicotinamide N-methyltransferase (NNMT) is the major NAM-metabolizing enzyme that catalyses the transfer of a methyl group from *S*-adenosylmethionine (SAM) to the ring nitrogen of NAM, yielding 1-methylnicotinamide (MNA) and *S*-adenosylhomocysteine (SAH) (Fig. 1). NNMT also regulates the biosynthesis of NAD^+^ by metabolizing NAM, which is a potent sirtuin inhibitor as well as a precursor for NAD^+ ^^3,11^. Thus, NNMT activation might influence sirtuin activities. In addition, NNMT activation affects the methylation capacity in tissues, where the tissue SAM/SAH ratio, an index of methylation capacity, decreases^12^. NNMT is overexpressed in many cancer cell lines, resulting in decreased SAM/SAH ratios and reduced gene methylation, which may contribute to tumorigenesis^13^. Hence, NNMT has emerged as a modulator of cell metabolism by connecting metabolic and epigenetic pathways.

NNMT is predominantly expressed in the liver^14^, suggesting a role in hepatic energy homeostasis or pathogenesis of hepatic steatosis. Consistently, NNMT gene variants may also serve as risk factors for the development of NASH^15^. Urinary and serum levels of MNA and its metabolite, 2-pyridone-5-carboxamide (2-PY), are increased in patients with cirrhosis, suggestive of increased NNMT activity associated with human liver cirrhosis^16^. Recent study also revealed that reducing NNMT expression, using an antisense oligonucleotide, may ameliorate high fat diet-induced adiposity and fatty liver^17^. However, the details in the mechanism for the involvement of NNMT in hepatic energy metabolism or hepatic steatosis have not been fully resolved.

In this study, we investigated the effects of NNMT on hepatic steatosis and fibrosis using transgenic (Tg) mice that overexpress NNMT. The NNMT Tg mice manifested fatty livers and fibrosis, especially after NAM supplementation. We further examined the roles of NAD^+^ and methionine pathways in these mice.

## Results

### The overexpression of NNMT induced steatosis in mice

**Figure 2:**
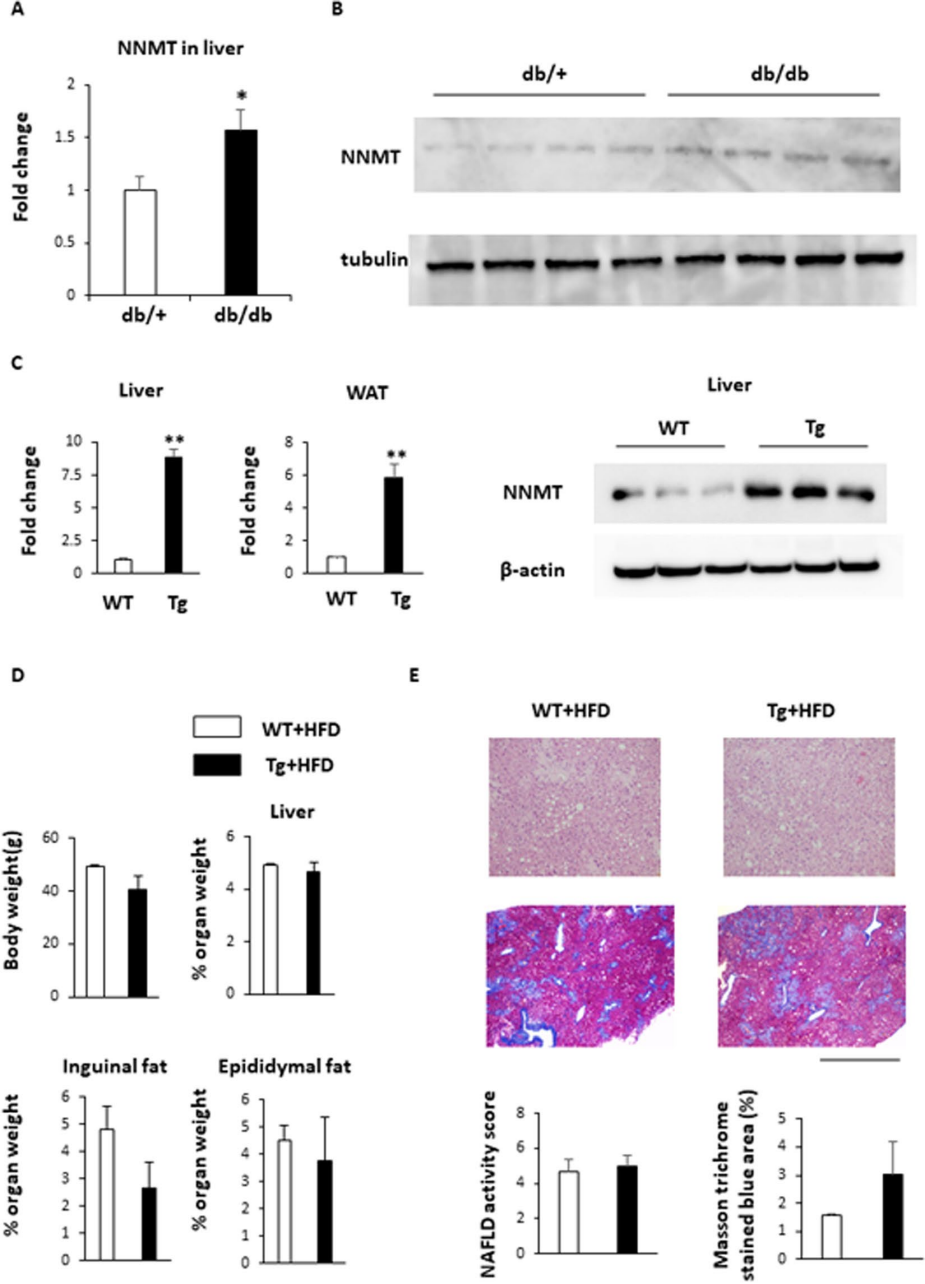
NNMT expression in the livers of obese mice and the generation of NMMT transgenic mice. (**A**) NNMT mRNA expression, normalized to 36B4 levels, in the livers of genetically obese (db/db) mice (black bars) and their normal (db/+) littermates (white bars) (n = 4, 5 per group). (**B**) NNMT protein levels in the livers of obese (db/db) and lean (db/+) mice. (**C**) NNMT mRNA expression levels in WAT and livers of Tg mice (black bars) and WT littermates (white bars) were measured by real-time PCR (n = 5 per group). NNMT protein levels in livers of Tg mice and WT mice were determined by Western blot analysis. (**D**) Body weight and liver, epididymal, and inguinal fat weight/body weight ratios, after being fed an HFD for 3 months, in Tg and WT mice (n = 3 per group). (**E**) H-E staining (upper panel) and Masson trichrome staining (lower panel) of liver from Tg and WT mice fed an HFD for 3 months. Bars represent 1.0 mm. NAFLD activity score and Masson trichrome stained blue area (%) were assessed (n = 3 per group). **P < 0.01 versus WT mice. Full-length blots/gels are presented in Supplementary Fig. 6. Abbreviations: NNMT, nicotinamide N-methyltransferase; WAT, white adipose tissue; PCR, Polymerase chain reaction; Tg, transgenic; WT, wild type; HFD, high fat diet.

**Figure 3:**
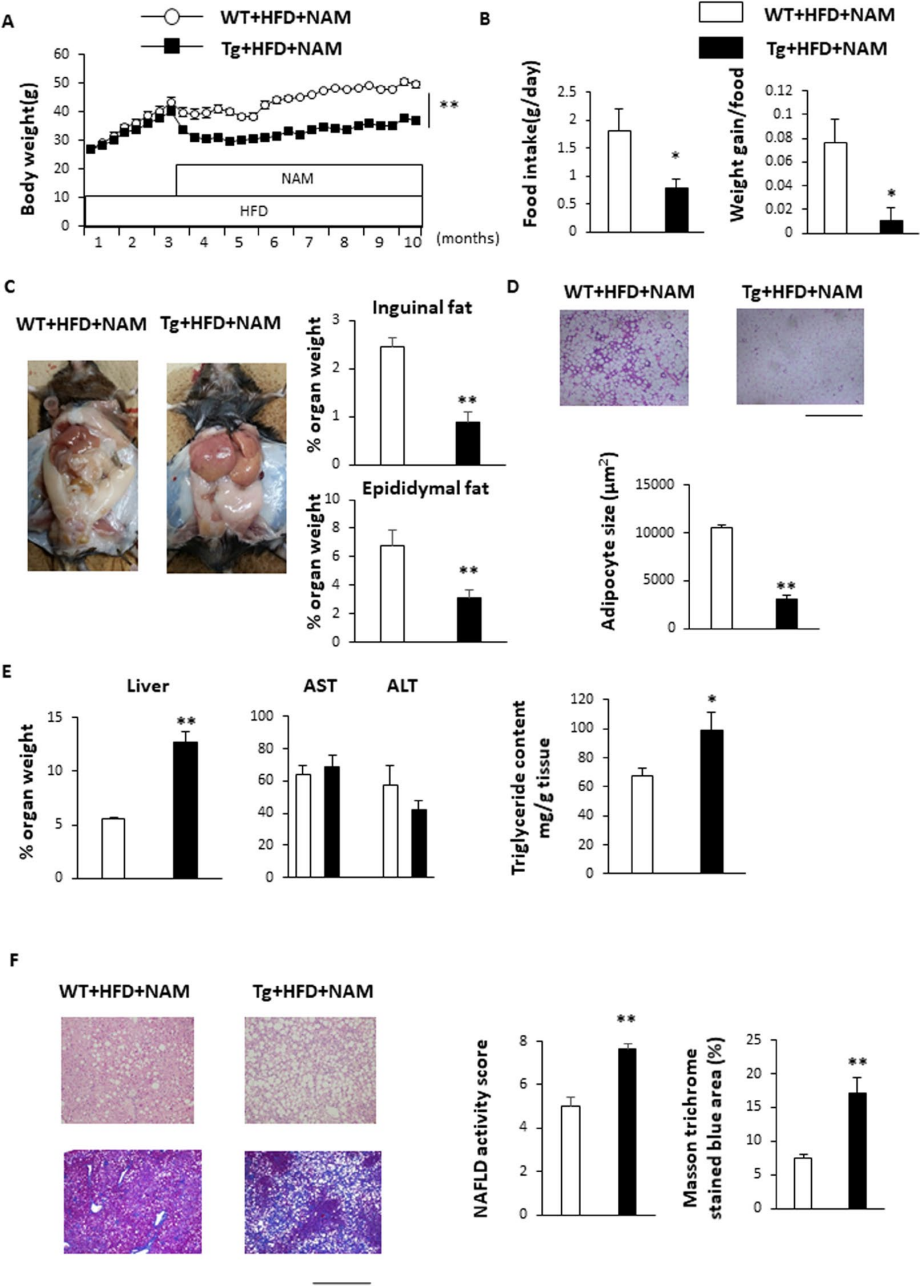
HFD with NAM induces hepatomegaly and liver fibrosis in male NNMT Tg mice. (**A**,**B**) After 3 months of HFD feeding, mice were treated with NAM. Body weight, food consumption, and food efficiency after administration of a NAM-supplemented HFD for 7 months in NMMT Tg mice (black bars) and WT littermates (white bars) (n = 5–8 per group) were measured. (**C**) Mouse abdominal cavity demonstrating hepatomegaly in Tg mice. Epididymal and inguinal fat weight/body weight ratios in Tg and WT mice (n = 5–6 per group) after being fed a NAM-supplemented HFD for 7 months. (**D**) Histology of epididymal adipose tissue in mice on an HFD with NAM. Mean adipocyte size in Tg versus WT mice (n = 4/group). (**E**) The bar graph shows the liver weight/body weight ratio and serum levels of AST and ALT, after being fed an HFD with NAM, in Tg and WT mice (n = 5–6 per group). Liver triglyceride content in Tg and WT mice after high-fat diet (n = 5–6 per group). (**F**) H-E staining (upper panel) and Masson trichrome staining (lower panel) of liver from Tg and WT mice fed an HFD with NAM for 7 months. Bars represent 1.0 mm. NAFLD activity score and Masson trichrome stained blue area (%) were assessed (n = 5–6 per group). *P < 0.05, **P < 0.01 versus WT mice. Abbreviations: NAM, nicotinamide; NNMT, nicotinamide N-methyltransferase; HFD, high fat diet; Tg, transgenic; WT, wild type.

NNMT expression was previously shown to be elevated in white adipose tissue (WAT) and in the livers of obese mice and humans^17^. We confirmed that this NNMT mRNA expression (Fig. 2A) as well as NNMT protein levels were significantly higher in livers from genetically obese (db/db) mice than in those from lean mice (Fig. 2B). To explore the pathological role of NNMT expression in the development of obesity and fatty liver, we generated Tg mice that overexpressed NNMT. The NNMT mRNA expression levels were 9-fold higher in the livers and 6-fold higher in the epididymal fat of Tg mice, compared with the levels in their wild-type (WT) littermates (Fig. 2C). The NNMT mRNA expression levels were also higher in brain, skeletal muscle, and heart of Tg mice (Supplement Fig. 1A). Liver NNMT protein level in Tg mice was higher than WT mice (Fig. 2C). We fed the Tg mice either a standard diet or a high fat diet (HFD) for 3 months. NNMT overexpression did not alter body weights, liver weights, adipose tissue weights, or hepatic lipid accumulation, regardless of whether the animals were fed a standard diet (Supplementary Fig. 1B,C) or an HFD (Fig. 2D,E). To further investigate the role of NNMT in hepatic metabolic changes, Tg mice were placed on an HFD containing NAM in order to enhance the NNMT activity. After 3 months of HFD feeding, mice were treated with NAM (Fig. 3A). When fed a NAM-supplemented HFD, the Tg mice were resistant to HFD-induced obesity, demonstrating significantly lower body weights than WT mice fed the same diet (Fig. 3A). The decrease in body weights were attributed, at least in part, to reduced food intakes and decreased food conversion efficiency evaluated by weight gain to food intake ratio (Fig. 3B), which are known effects caused by NAM^18^. Next, NNMT Tg mice and WT mice were fed a HFD either with or without NAM for 8 months (Supplementary Fig. 2A). NAM resulted in significant decrease in body weight in WT mice as previously reported^18^, and further decreased body weight in Tg mice on HFD (Supplementary Fig. 2A). Thus, these results indicated that the effect of NAM were augmented in NNMT Tg mice. In accordance with the observed decrease in body weight, the weights of the inguinal and epididymal adipose tissue masses were significantly lower in the Tg mice than in the WT animals (Fig. 3C and Supplementary Fig. 2B) and the adipocytes were also smaller in the Tg mice (Fig. 3D). Despite the observed reductions in body weight and fat mass, the Tg mice manifested hepatomegaly, unlike the WT mice (Fig. 3C,E and Supplementary Fig. 2B). Serum AST and ALT levels were similar between the Tg and WT mice fed a NAM-supplemented HFD (Fig. 3E). Histological examination showed significant increased accumulation of hepatic triglyceride content, NAFLD activity score and fibrosis (Fig. 3F and Supplementary Fig. 2C). Although body and WAT weights were comparable between the Tg and WT mice (Supplementary Fig. 3A,B), female Tg mice on an HFD with NAM supplementation demonstrated higher liver weights and NAFLD activity score compared with female WT mice on the same diet (Supplementary Fig. 3C). These data indicate that NNMT combined with NAM supplementation contributes to the development of hepatic steatosis and fibrosis in mice, independent of the effects on body weight.

### The effects of NNMT overexpression on lipid metabolism

**Figure 4:**
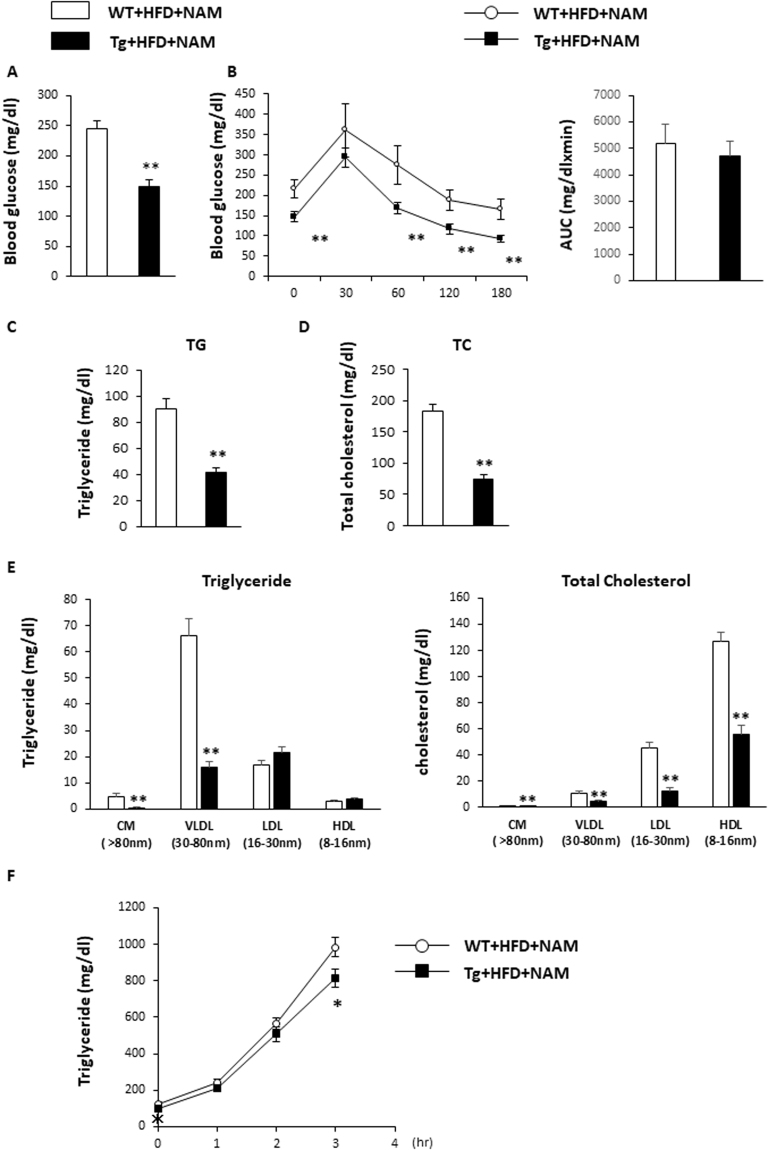
NNMT Tg mice have improved lipid metabolism. (**A**) Fasting blood glucose levels of NMMT Tg mice and WT littermates fed a NAM-supplemented HFD for 7 months (n = 6–9 per group). (**B**) Glucose tolerance testing and the area under the curve (AUC) for glucose in Tg and WT mice (n = 5–8 per group). Plasma triglyceride (**C**) and total cholesterol (**D**) concentrations were determined in fasted Tg and WT mice (n = 5–6 per genotype). (**E**) Lipoprotein profiles of plasma triglycerides (left panel) and cholesterol (right panel), after fasting (n = 4 per genotype). (**F**) Triglyceride concentrations following triton injection in fasted Tg and WT mice (n = 6–9 per group). *P < 0.05, **P < 0.01 versus WT mice. Abbreviations: NAM, nicotinamide; NNMT, nicotinamide N-methyltransferase; HFD, high fat diet; Tg, transgenic; WT, wild type.

Fasting blood glucose levels were significantly lower in Tg mice on a NAM-supplemented HFD, compared with WT mice on the same diet (Fig. 4A). Blood glucose concentrations were also significantly lower at 0, 60, 120, and 180 min after a glucose challenge in Tg mice than in WT mice (Fig. 4B). However, area under the curve of the glycemic response was not different between two groups, indicating glucose disposal in these mice was similar (Fig. 4B). Fasting total cholesterol and triglyceride levels were lower in the Tg mice than in the WT mice (Fig. 4C,D). Consistent with the observed low triglyceridaemia, very low density lipoprotein (VLDL) levels were lower in Tg mice than in WT mice, as determined by high-performance liquid chromatography (HPLC; Fig. 4E). The liver VLDL secretion rates were also lower in Tg mice (Fig. 4F). These data indicated that NNMT overexpression affects lipid metabolism.

### The effects of NNMT overexpression on gene expressions involved in hepatic steatosis, inflammation, and fibrosis

**Figure 5:**
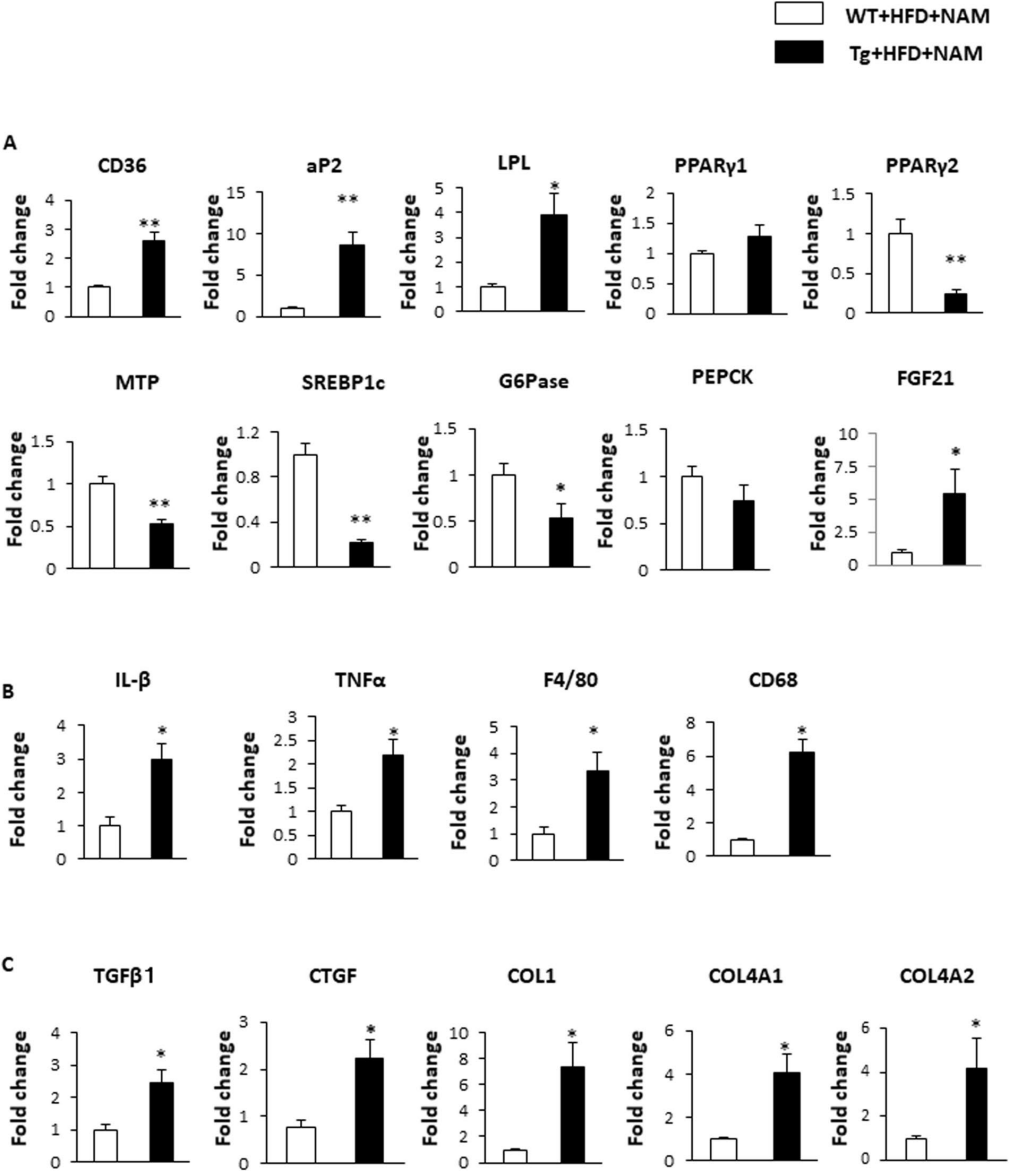
NNMT overexpression induces genes involved in FFA uptake, VLDL secretion, inflammation, and fibrosis in the liver. Total RNA samples, isolated from the livers of NMMT Tg mice (black bars) and WT littermates (white bars) on an HFD with NAM for 7 months, were analysed using real-time RT-PCR. The amounts of each mRNA related to hepatic lipid metabolism (**A**), tissue inflammatory responses (**B**), and tissue fibrosis (**C**) were measured and normalised to β-actin mRNA levels. The values shown are the means ± SEM (n = 3–6 per group). *P < 0.05, **P < 0.01 versus WT mice. Abbreviations: NAM, nicotinamide; NNMT, nicotinamide N-methyltransferase; HFD, high fat diet; Tg, transgenic; WT, wild type; FFA, free fatty acid; VLDL, very low density lipoprotein; LPL, lipoprotein lipase; PPAR, peroxisome proliferator-activated receptor; MTP, microsomal triglyceride-transfer protein; SREBP1c, sterol regulatory element-binding protein 1c; G6Pase, glucose-6 phosphatase; PEPCK, phosphoenolpyruvate carboxykinase; FGF21, fibroblast growth factor 21; IL, interleukin; TNF-α, tissue necrosis factor-α; TGFβ1, transforming growth factor β1; CTGF, connective tissue growth factor; COL, collagen.

The expression of the genes involved in the pathogenesis of fatty liver and liver fibrosis was compared between Tg and WT mice fed a NAM-supplemented HFD. The expression levels of genes related to lipid uptake, such as cluster of differentiation (CD) 36, aP2, and lipoprotein lipase, were significantly higher in Tg mice than in WT mice (Fig. 5A). Although these genes are reported to be peroxisome proliferator-activated receptor (PPAR)γ targets, *PPARγ1* and *PPARγ2* expression levels were not elevated in Tg mice (Fig. 5A). In accordance with the observed reduced VLDL secretion (Fig. 4F), microsomal triglyceride-transfer protein expression significantly was decreased in Tg mice (Fig. 5A). The expression levels of genes related to hepatic lipogenesis (e.g., *SREBP1c*) were decreased. Regarding glucose metabolism, glucose-6-phosphate expression was significantly decreased in Tg mice, compared with WT mice. The expression of fibroblast growth factor 21 (FGF21), a humoral factor secreted from the liver and involved in energy metabolism, was expressed at levels 5-fold higher in Tg mice than in WT mice (Fig. 5A). We also examined genes related to hepatic inflammation and fibrosis in liver tissue. Inflammatory cytokines (e.g., interleukin [IL]-1β and tissue necrosis factor-alpha [TNFα]) and macrophage makers (e.g., F4/80 and CD68) were significantly upregulated in Tg mice (Fig. 5B), compared to WT mice. The genes involved in extracellular matrix regulation, such as those encoding transforming growth factor β (TGF-β), connective tissue growth factor (CTGF), collagen (COL) 1, COL4A1, and COL4A2 were also elevated in Tg mice, compared with WT mice (Fig. 5C).

### The effects of NMMT overexpression on hepatic NAD^+^ content

**Figure 6:**
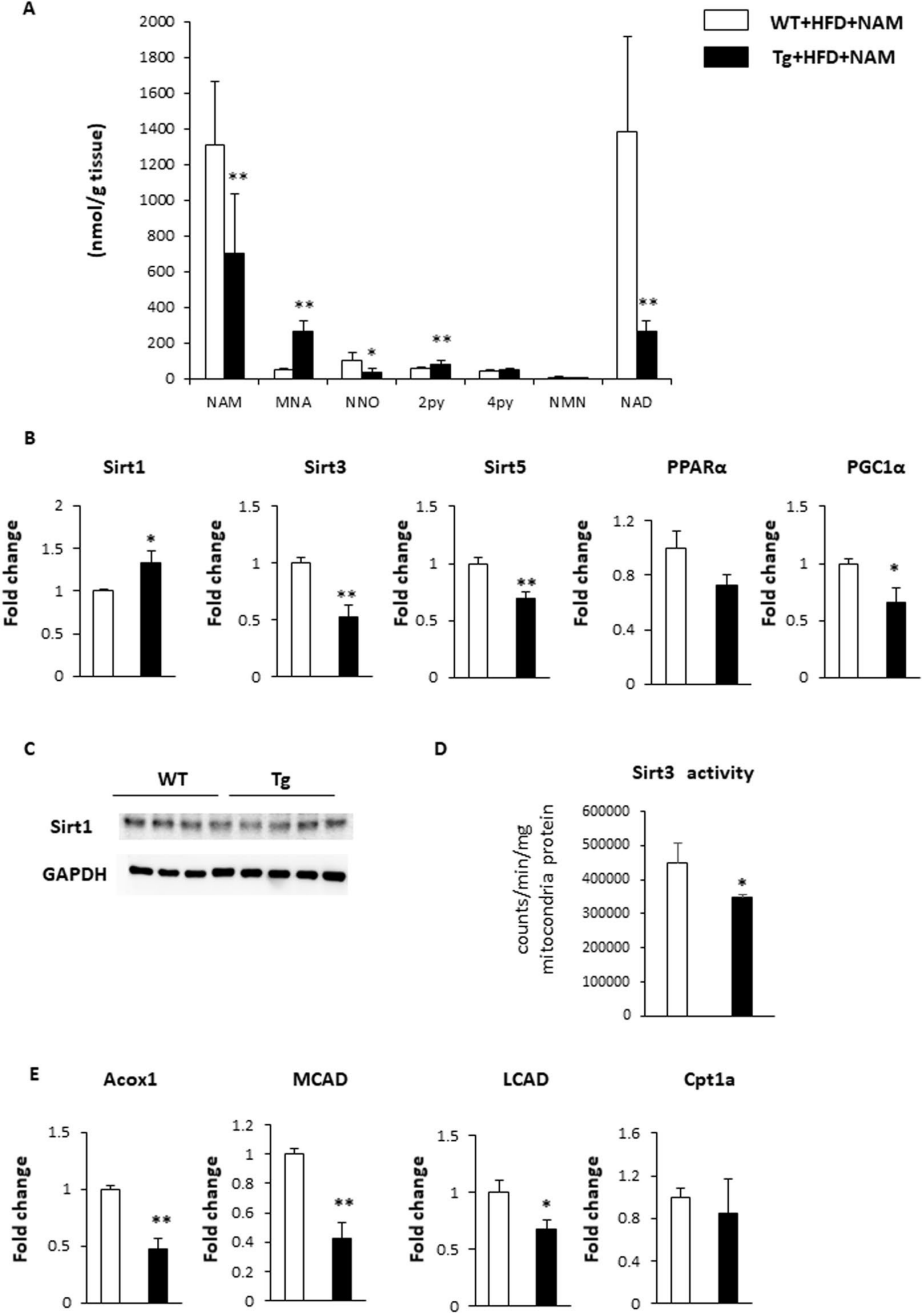
NNMT overexpression alters nicotinic acid metabolites and affects Sirt3/PGC-1α target genes. (**A**) Nicotinic acid metabolites in the livers of NMMT Tg mice (black bars) and WT littermates (white bars) on an HFD with NAM for 7 months. The data represent the means ± SEM (n = 5–6 animals/group). (**B**) Relative mRNA expression levels in the livers of Tg mice (black bars) and WT mice (white bars) on an HFD with NAM. (n = 5–6 per group). *P < 0.05, **P < 0.01 versus WT mice. (**C**) Sirt1 protein expression levels in the livers of Tg mice and WT mice on an HFD with NAM for 7 months. (**D**) Sirt3 activity in livers of Tg mice (black bars) and WT mice (white bars) on an HFD with NAM for 7 months. (n = 5–6 per group). *P < 0.05 versus WT mice. (**E**) Relative mRNA expression levels in the livers of Tg mice (black bars) and WT mice (white bars) on an HFD with NAM. (n = 5–6 per group). *P < 0.05, **P < 0.01 versus WT mice. Full-length blots/gels are presented in Supplementary Fig. 6. Abbreviations: NAM, nicotinamide; MNA, 1-methylnicotinamide; NNO, nicotinamide-N-oxide; 2PY, N-methyl-2-pyridone-5-carboxamide; 4PY, N-methyl-4-pyridone-5-carboxamide; NMN, nicotinamide mononucleotide; NAD+, nicotinamide adenine dinucleotide; HFD, high fat diet; Tg, transgenic; WT, wild type; sirt, sirtuin; PPAR, peroxisome proliferator-activated receptor; PGC-1α, peroxisome proliferator-activated receptor-gamma coactivator 1 α; Acox1, acyl-coenzyme A oxidase 1; MCAD, medium chain acyl coenzyme A dehydrogenase; LCAD, very long chain acyl coenzyme A dehydrogenase; Cpt1a, carnitine palmitoyltransferase 1A.

We measured nicotinic metabolite levels in the livers of Tg and WT mice on a NAM-supplemented HFD. In the livers of Tg mice, hepatic NAM and NAD^+^ levels were significantly lower than in WT mice. In contrast, the downstream products of NNMT, such as MNA and 2PY, accumulated in the livers of Tg mice (Fig. 6A). We observed similar nicotinic metabolite change among 4 groups (Supplementary Fig. 4A). Since Sirtuin activity can be increased by NAD^+^ concentration and supressed by NAM, the NAD^+^/NAM ratio may be a better predictor of Sirtuin activity^3^. We observed that the NAD^+^/NAM ratio was reduced from 1.0 to 0.3–0.5 in Tg mice. Among the several isoforms of sirtuins, Sirt1, Sirt3, and Sirt5 are considered to be the key NAD^+^ sensors^3^. The expressions of these sirtuins and the related downstream target genes that are involved in the pathogenesis of fatty liver were examined. Although *Sirt1* mRNA expression marginally increased, Sirt1 protein expression was not different between WT and Tg mice (Fig. 6B,C). *Sirt3* and *Sirt5* expressions were significantly decreased in NNMT Tg mice (Fig. 6B), which implied the decreased activities of Sirt3 and Sirt5 in Tg mice. Indeed, Sirt3 activity was significantly reduced in the Tg mice (Fig. 6D). Sirt3 increases fatty-acid oxidation directly as well as indirectly through the induction of peroxisome proliferator-activated receptor gamma coactivator 1-alpha (PGC-1α)^19,20^. Consistently, PGC-1α expression and the genes involved in fatty acid oxidation including acyl-coenzyme A oxidase 1 (Acox1), medium-chain acyl-CoA dehydrogenase (MCAD) and long-chain acyl-CoA dehydrogenase (LCAD) were reduced in Tg mice, compared with WT mice (Fig. 6B,E). These data indicate that changes in nicotinic metabolite levels contribute to the development of hepatic steatosis, in part, via the Sirt3/PGC-1α pathway.

### The effects of NMMT overexpression on hepatic SAM and SAH production

**Figure 7:**
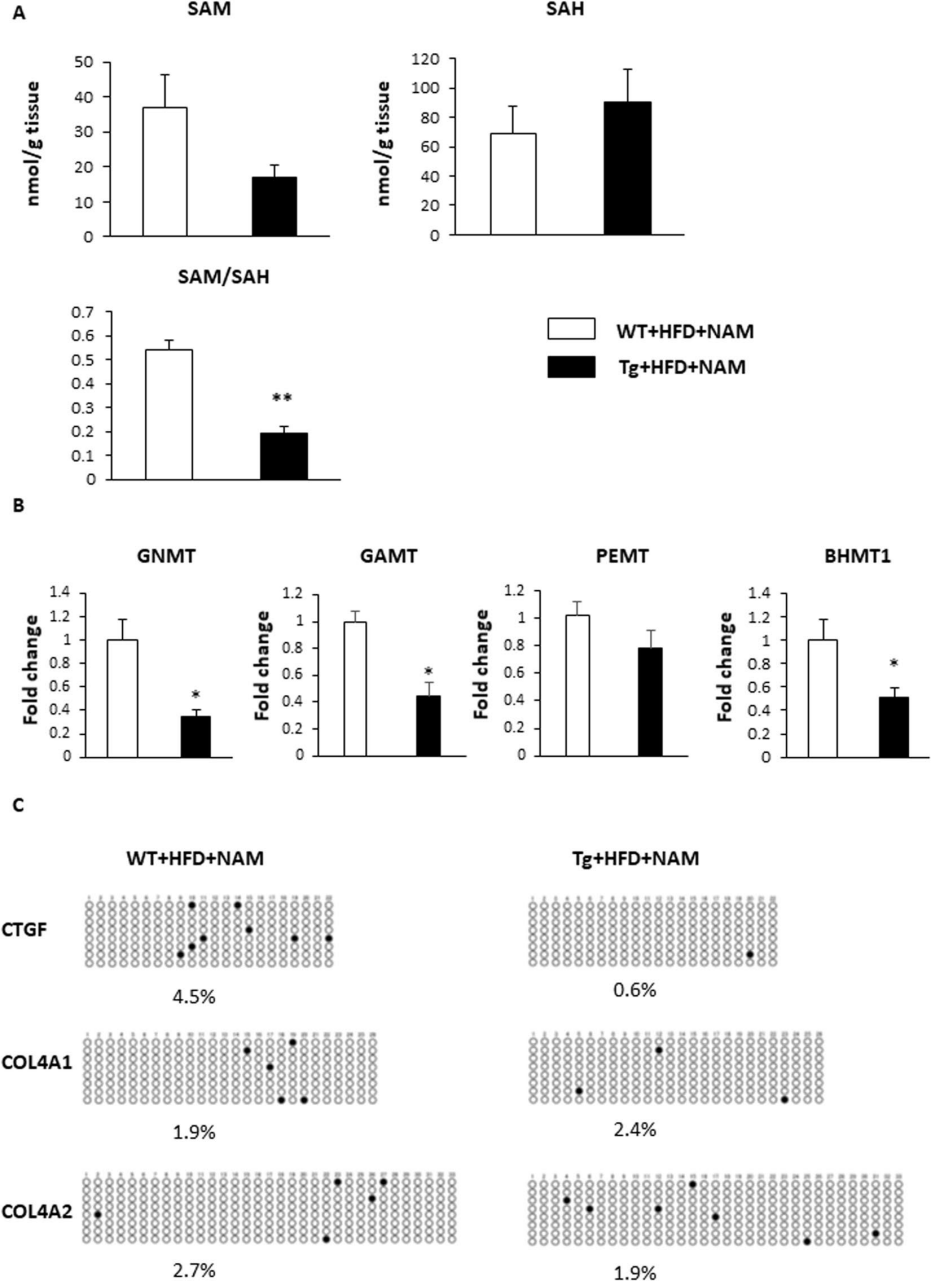
NNMT overexpression reduces the liver SAM/SAH ratio and decreases the level of CTGF promoter methylation. (**A**) SAM levels, SAH levels, and SAM/SAH ratios in the livers of NMMT Tg mice (black bars) and WT littermates (white bars) on an HFD with NAM. The data represent the means ± SEM (n = 3 animals/group). (**B**) Relative GNMT, GAMT, PEMT and BHMT1 mRNA expression in the liver. (n = 3 per group). (**C**) Bisulfite sequencing of the CTGF, Col4a1 and Col4a2 CpG island. Each circle indicates a CpG site in the sequence, and each line of circles represents analysis of a single cloned allele. ○, unmethylated CpG sites; •, methylated CpG sites. Abbreviations: NNMT, nicotinamide N-methyltransferase; SAM, S-adenosylmethionine; SAH, S-adenosylhomocysteine; HFD, high fat diet; NAM, nicotinamide; Tg, transgenic; WT, wild type; GNMT, glycine N-methyltransferase; GAMT, guanidinoacetate N-methyltransferase; PEMT, phosphatidylethanolamine N-methyltransferase; BHMT, betaine homocysteine methyltransferase; MSP, methylation-specific polymerase chain reaction; CTGF, connective tissue growth factor; COL, collagen.

NNMT is reported to regulate SAM and SAH levels (Fig. 1)^13^. Hepatic SAM levels were lower and SAH levels were higher in Tg mice on a NAM-supplemented HFD (Fig. 7A). The SAM/SAH ratio, an indicator of methylation capacity^12^, in liver is typically 3–5. However, our data of the SAM/SAH ratio in livers of WT mice with NAM was below 1, probably reflecting that NAM administration reduced methyl-group deficiency and SAM/SAH ratio^18,21^. Moreover, the SAM/SAH ratio was significantly lower in Tg mice on a NAM-supplemented HFD (Fig. 7A). Among the genes related to methionine metabolism, we examined the expression of glycine N-methyltransferase (*GNMT*), guanidinoacetate N-methyltransferase (*GAMT*), and phosphatidylethanolamine N-methyltransferase (*PEMT*), the main methyltransferases mediating SAM catabolism in the liver^22^, and betaine homocysteine S-methyltransferase 1 (*BHMT1*), which catalyses the re-methylation of homocysteine to generate methionine^23^ (Fig. 1). The hepatic expression levels of *GNMT, GAMT* and *BHMT1* in Tg mice were significantly lower than in WT mice (Fig. 7B). These data indicate that NNMT overexpression, combined with decreased re-methylation, depletes the methyl pool and dampens the methylation cycle in the liver. A reduced methylation capacity has been reported to promote the development of fatty liver and steatohepatitis by affecting gene methylation^24^. We examined the methylation status of the CTGF, COL4A1, and COL4A2 CpG island because we observed that these genes were highly induced in Tg mice (Fig. 5C) and demethylation of the CpG island of these genes increases the expression^25–29^. Bisulfite sequencing result of CTGF demonstrated significant decreased DNA methylation in Tg mice compared with WT mice (Fig. 7C). The hypomethylation status of COL4A1 and COL4A2 were comparable between the Tg and WT mice (Fig. 7C), suggesting that the effect of NNMT overexpression on hypomethylation status of fibrosis related genes are gene specific. These results indicated that NNMT induces *CTGF* expression, at least in part, via an epigenetic mechanism, which contributes to the pathogenesis of liver fibrosis.

## Discussion

NNMT has been recently reported to modulate energy expenditures in adipose tissue^11^. However, its role in the liver has not been elucidated. In the present study, we demonstrated that NNMT, together with NAM supplementation, promotes hepatic steatosis and fibrosis, despite increased lipid metabolism and reduced adiposity. NNMT overexpression induced the genes important for hepatic steatosis and fibrosis by reducing the tissue NAD^+^ content and methylation pool, indicating that NNMT links NAD^+^ and methionine metabolism and contributes to the progression of fatty liver disease.

NA and NAM are precursors of NAD^+^ biosynthesis that have potentially beneficial effects on metabolic disorders resulting from their ability to activate Sirtuins^3^. However, high doses of NA or NAM can also induce hepatic injury, which limits their practical use^6–8^ and high dose of NAM serves as a Sirtuin inhibitor^11^. Although NAM treatment inhibited HFD-induced obesity in this study (Supplement Fig. 2A,B), it had a marginal effect on liver in WT mice (Supplement Fig. 2B,C). This is probably because the effect of NAM on liver was cancelled out by continuous decreased food intake and body weight in NAM treatment (Supplement Fig. 2A). NNMT overexpression, in conjunction with elevated NAM levels, augments hepatic steatosis by inhibiting Sirt3 activity (Fig. 6). In our experiments, the NAD^+^/NAM ratio decreased from 1.0 to 0.3 in Tg mice, possibly accounting for the decreased Sirt3 expression and the inhibition of fatty acid oxidation. Recently, MNA has also been shown to increase Sirt1 activity by stabilizing the protein^30^. However, in this study, the hepatic Sirt1 protein expression was not different between WT and Tg mice (Fig. 6C). Further, the hepatic Sirt1 target, PPARα, and its target genes were unchanged (Fig. 6B, Supplement Fig. 5)^31^. The hepatic MNA concentration was relatively low compared with the NAM and NAD^+^ concentrations, the role of MNA may be unimportant in our mouse model.

NNMT is known for the coupling NAD^+^ metabolism with methionine cycle (Fig. 1). Hong S *et al*. reported that NNMT knockdown with adenovirus did not alter methionine cycle (the ratio of SAM/SAH) nor NAD^+^ content in the liver^30^. We measured NA metabolites in both NNMT Tg mice and WT mice under HFD ± NAM (Supplement Fig. 4A). NAD^+^ metabolite in liver is not significantly different between the Tg mice and WT mice under HFD without NAM. In this regard, it is considered that NNMT is not a dominant methyltransferase in the liver^32^. GAMT, GNMT, and PEMT are thought to be major methyltransferases. Therefore, overexpression of NNMT is likely not sufficient to change liver NAD^+^ metabolism/methyl donor balance under HFD. However, under NAM-replete condition, such as under NAM rich food intake^33^, NNMT play a crucial role in the NAD + metabolism and methionine cycle as expected from our results. In this regard, increased NNMT expression considered to be of clinical relevance.

NAM is a substrate for both NNMT and nicotinamide phosphoribosyltransferase (NAMPT, Fig. 1)^30^. Our data suggested that when NAM, an NMMT substrate, was abundantly available, NNMT activation drove the depletion of both NAD^+^ and SAM, leading to liver steatosis and fibrosis. From standpoint of clinical nutrition, NAM intake has increased over the years as a result of the shift towards a more meat-centric diet^33^, and intake of NAM and NNMT activity has been suggested to be augmented in obese people^17,34^. Thus, our findings uncovered the potential involvement of NNMT in the pathogenesis of fatty liver and hepatic fibrosis.

Our study revealed the molecular changes involved in the pathogenesis for fatty liver and hepatic fibrosis by the overexpression of NMMT with NAM supplementation. It is generally accepted that the aberrant regulation of fatty acid and cholesterol metabolism in the liver contributed to the hepatic lipid accumulation. Some of the gene alteration related to the fatty acid and cholesterol metabolism can be explained by the reduced expression and activity of Sirt3 in the liver. Recent studies revealed that Sirt3 regulates mitochondrial function such as fatty acid oxidation and oxidative stress^19,35^, and has an important role in the steatohepatitis. Sirt3 deficient mice are vulnerable to methionine-choline deficient diet-induced NASH model by increasing oxidative stress and manifest hepatic inflammation and fibrosis^36^, as seen in the Tg mice with NAM supplementation. Moreover, NNMT overexpression with high dose of NAM could compete with other methyltransferase reactions for SAM resulting in the downregulation of these methyltransferases (Figs 1 and 7). Other methyltransferase deficient mice such as GNMT manifest similar phenotypes with Tg mice^22^. Therefore, Sirt3 and functional other methyltransferases deficiency seem to be a key enzyme for deterioration of NASH in our mice model.

Supplementing the diet with methyl donors, such as choline or methionine were reported to prevent fatty livers in rats fed an HFD supplemented with either NA^37^ or NAM^18^. Additionally, methylation deficiency-induced liver injury is associated with epigenetic gene modification^21^, with promoter hypomethylation often associated with transcriptional permissiveness. Further, livers with advanced NAFLD show increased expression of hypomethylated genes, compared with livers showing only mild NAFLD^38^. Thus, the genes involved in producing matrix components and remodelling factors, such as *COL1a1*, *COL1A2*, *COL4A1*, and *CTGF*, are hypomethylated and show increased expression^38^. Among these genes, *CTGF* is the main contributor to liver fibrosis^39^. Here, we demonstrated that, consistent with the decrease in methyl donor capacity, the *CTGF* promoter is hypomethylated and that increased *CTGF* expression occurred in NNMT Tg mice (Figs 5C and 7C). Therefore, NNMT, in conjunction with a NAM-rich diet, aggravates liver fibrosis, at least in part, through epigenetic changes to *CTGF*.

Body weight and adiposity decreased in Tg mice fed an HFD with supplemental NAM. In humans, NA has a weight loss effect^7^, and treatment with NAM can also decrease body weight by suppressing food intake and food conversion efficiency through unknown mechanisms^18^. In this study, the effects of NAM on body weight and food intake were augmented in male Tg mice (Fig. 3 and Supplement Fig. 2). The induction of fibroblast growth factor (FGF) 21, a metabolic regulator expressed mainly in the liver, may also contribute to reduced adiposity. FGF21 acts via the activation of brown adipose tissue and the browning of WAT, leading to increased glucose uptake and energy metabolism^40^. Methionine-choline deficient diet increases hepatic FGF21 expression^41^. Decreased expression of *PGC-1α* (Fig. 6B) might also upregulate FGF21 expression, further enhancing weight loss and reduced adiposity^42^. Energy expenditure changes might contribute to the body weight reductions in male NNMT Tg mice (Fig. 3A,B), and increased NNMT expression in metabolite tissues such as brain, skeletal muscle, and heart (Supplement Fig. 1A) might also affect metabolic phenotype seen in Tg mice. However, the details of energy expenditures in these mice require further investigation.

In conclusion, we demonstrated that NNMT overexpression in mice administered a NAM-supplemented HFD promoted hepatic steatosis and fibrosis. Because NNMT is a potent regulator of NAD^+^ metabolism and epigenetic modifications in the liver, our data suggest a potentially novel strategy for blocking the progression to NASH by inhibiting NNMT function.

## Materials and Methods

### Materials and experimental ethics

NAM, β-NAM mononucleotide, and β-NAM adenine dinucleotide sodium salt were purchased from Sigma-Aldrich (St. Louis, MO, USA). Nicotinic acid, perchloric acid, and phosphoric acid were purchased from Wako Pure Chemical Industries (Osaka, Japan). We obtained 1-methylnicotinamide chloride from Santa Cruz Biotechnology (Dallas, TX, USA). NAM N-oxide and formic acid were purchased from Nacalai Tesque (Kyoto, Japan). N-methyl-2-pyridone-5-carboxamide and N-methyl-4-pyridone-5-carboxamide were purchased from Toronto Research Chemicals (Toronto, ON, Canada). Methanol and acetonitrile were purchased from Kanto Chemical (Tokyo, Japan). This study was performed in accordance with the institutional guidelines of the Animal Care and Experimentation Committee at Keio University (Tokyo, Japan), which also approved the experimental protocols (Approval No. 15010). All surgeries were performed under sodium pentobarbital anaesthesia, and all efforts were made to minimize animal suffering.

### Generation and identification of mice expressing NNMT

NNMT cDNA was subcloned into the pCAG-cDNA plasmid. The expression cassette, including the CAG promoter, was excised from the construct and injected into pronuclear-stage embryos of C57BL/6 J mice (Unitech, Chiba, Japan). The transgene copy number was determined using Southern blot analysis of DNA from the tails of the animals, and the transgenic lines were maintained by backcrossing to C57BL/6 mice.

### NAM treatment

NAM supplementation was accomplished by providing the mice with drinking water containing 1% NAM^43^. A dose of 1% NAM has no apparent carcinogenic effect^44^. A 0.1% NAM dose is approximately equivalent to 100 mg/kg daily^45^. In this study, a dose of 1% NAM in drinking water for HFD was approximately equivalent to 600 mg/kg BW/day. Obesity was induced using a HFD (45% calories from fat; D12451, Research Diets Inc.), beginning at 6 weeks of age. The HFD used in this study contains 30 mg/kg (diet) nicotinic acid. Nicotinic acid is metabolized to NAD^+^ or NAM (Fig. 1). Therefore, each HFD-fed mouse consumed around only 1 mg/kg BW/day nicotinic acid, which can be additional NAM intake. For the experiment shown in Fig. 3, mice were treated with NAM for 7 months after 3 months of HFD feeding. For the experiment shown in Supplementary Figure 2, mice were fed a HFD either with or without NAM for 8 months

### Oral glucose tolerance test

Oral glucose tolerance tests were conducted on animals following a 6-h fasting period. A bolus of glucose (2 g/kg body weight) was delivered into the stomach by a feeding needle. Blood glucose levels were measured at 0, 30, 60, 120, and 180 min after glucose gavage with a portable glucose meter (Glu-test Sensor; Sanwa) by tail snipping. Area under the curve of the glycemic response was calculated after subtracting the fasting blood glucose levels from 0 to 60 min after glucose administration.

### Hepatic VLDL production

After overnight fasting, mice were injected intraperitoneally with Poloxamer 407 (1 g/kg body weight), which inhibits lipoprotein clearance^46^. Blood was sampled before (0 hours) and after (1 and 2 hours) injection. Plasma was separated, and triglyceride levels were determined enzymatically using the L-Type TG Kit (Wako Pure Chemicals).

### Lipoprotein profiling

Fasting blood samples were drawn by retro-orbital bleeding, and plasma was acquired by centrifugation and stored at −20 °C until measurement. Plasma lipoproteins were analysed using HPLC (Skylight Biotech, Akita, Japan)^47^. Briefly, lipoprotein fractions were defined based on lipoprotein particle diameters. Then, cholesterol and triglyceride levels of lipoprotein fractions (chylomicron (>80 mm), VLDL (30–80 nm), LDL (16–30 nm), and HDL (8–16 nm)) were measured by a dual detection HPLC system consisting of two tandem connected TSKgel Lipopropak XL columns (300 × 7.8 mm; Tosoh, Tokyo, Japan) (Skylight Biotech, Akita, Japan).

### Histological analysis

Formalin-fixed livers embedded in paraffin wax were sectioned and stained with hematoxylin and eosin (H-E) or Masson’s trichrome stains for histological analyses. The paraffin-embedded tissues were cut into sections of 3 *μ*m thickness, heated in an incubator at 46 °C for overnight, deparaffinized and hydrated. The serial sections were then stained for H-E and Masson’s trichrome as per standard protocols. Liver steatosis, inflammation, and ballooning were evaluated based on NAFLD activity score (NAS). Briefly, the proposed NAS was quantified by summing scores of steatosis (0–3), lobular inflammation (0–2), and hepatocellular ballooning (0–2)^48^. To quantify liver fibrosis, the extent of collagen deposition (blue area) in Masson’s trichrome stain were quantified using image-analysing software (the Image-Pro). Five fields were selected randomly from each mouse. The area of adipocytes was traced and quantified in 300 cells per mouse using the Image-Pro.

### Tissue lipid content

Lipid extraction was performed using the Bligh-Dyer method as described previously^49^. Briefly, liver tissues were homogenized in chloroform/methanol/H_2_O (1:2:0.8 v/v/v; room temperature), and were vortexed for 10–15 min. The phases were separated by addition of chloroform-water (final solvent ratio, chloroform/methanol/H_2_O, 1:1:0.9 v/v/v) and centrifugation at 4000 rpm for 15 min. An aliquot of the organic phase was collected, dried under nitrogen, and resuspended in 90% isopropanol + 10% Triton X-100. The triglyceride concentration in this aliquot was determined enzymatically with commercial kit (TG Kit; Wako Chemicals).

### Real-time PCR

Total RNA was extracted from mouse liver tissues using the TRIzol reagent. Equal amounts (1 µg) of total RNA from each sample were converted to cDNA using the PrimeScript RT Reagent Kit with gDNA Eraser (TaKaRa, Ohtsu, Japan), in 20-µL reaction volumes. Real-time PCR was performed using an ABI Step One Plus Real-Time PCR system (PE Applied Biosystems, Tokyo, Japan), with the amplification program set at 95 °C for 3 min, followed by 40 cycles of 95 °C for 10 s, 62 °C for 10 s, and 72 °C for 10 s. Sense and antisense cDNA primers, respectively, were as follows: ***NNMT***, 5′-AGGAACCAGGAGCCTTTGACT-3′, 5′-CCTGAGGGCAGTGCGATAGG-3′; ***Cd36***, 5′-GGCCAAGCTATTGCGACAT-3′, 5′-CAGATCCGAACACAGCGTAGA-3′; *aP2*, 5′-TGGAAGCTTGTCTCCAGTGA-3′, 5′-AATCCCCATTTACGCTGATG-3′; ***Lpl***, 5′-GTGGCCGAGAGCGAGAAC-3′, 5′-AAGAAGGAGTAGGTTTTATTTGTGGAA-3′; ***Pparγ1***, 5′-GTGAACCACTGATATTCAGGACATTT-3′, 5′-CCACAGAGCTGATTCCGAAGT-3′; ***Pparγ2***, 5′-AACTCTGGGAGATTCTCCTGTTGA-3′, 5′-TGGTAATTTCTTGTGAAGTGCTCATA-3′; ***MTP***, 5′-TGAGCGGCTATACAAGCTCAC-3′, 5′-CTGGAAGATGCTCTTCTCGC-3′; ***SREBP1c****,* 5′-GGAGCCATGGATTGCACATT-3′, 5′-GGCCCGGGAAGTCACTGT-3′; ***G6Pase****,* 5′-ACACCGACTACTACAGCAACAG-3′, 5′-CCTCGAAAGATAGCAAGAGTAG-3′; ***PEPCK***, 5′-CTTCTCTGCCAAGGTCATCC-3′, 5′-TTTTGGGGATGGGCAC-3′; ***FGF21****,* 5′-AGATCAGGGAGGATGGAACA-3′, 5′-TCAAAGTGAGGCGATCCATA-3′; ***IL-β***, 5′-GATTCTTTCCTTTGAGGCCCA-3′, 5′-ACAGAATATCAACCAACAAGTGATATTCTC-3′; ***TNF-α***, 5′-ACGTGGAACTGGCAGAAGAG-3′, 5′-CTCCTCCACTTGGTGGTTTG-3′; ***F4/80***, 5′-GCAAGGAGGACAGAGTTTATCGTG-3′, 5′-CTTTGGCTATGGGCTTCCAGTC-3′; ***CD68***, 5′-CAAGGTCCAGGGAGGTTGTG-3′, 5′-CCAAAGGTAAGCTGTCCATAAGGA-3′; ***TGFβ1***, 5′-GAAAGCCCTGTATTCCGTCTCCTT-3′, 5′-CAACAATTCCTGGCGTTACCTTGG-3′; ***CTGF***, 5′-GGGCCTCTTCTGCGATTTC-3′, 5′-ATCCAGGCAAGTGCATTGGTA-3′; ***COL1***, 5′-TCACCTACAGCACCCTTGTGG-3′, 5′-CCCAAGTTCCGGTGTGACTC-3′; ***Col4a1***, 5′-GGCGGTACACAGTCAGACCAT-3′, 5′-GGAATAGCCGATCCACAGTGA-3′; ***Col4a2***, 5′-GACCGAGTGCGGTTCAAAG-3′, 5′-CGCAGGGCACATCCAACTT-3′; ***Sirt1***, 5′-TCCTCACTAATGGCTTTCATTCCTG-3′, 5′-GTGCCAATCATGAGATGTTGCTG-3′; ***Sirt3***, 5′-TGCCAGCTTGTCTGAAGCA-3′, 5′-GTCCACCAGCCTTTCCACA-3′; ***Sirt5***, 5′-CTCCGGGCCGATTCATTTCC-3′, 5′-GCGTTCGCAAAACACTTCCG-3′; ***PPARα***, 5′-AGGAAGCCGTTCTGTGACAT-3′, 5′-AATCCCCTCCTGCAACTTCT-3; ***PGC1α***, 5′-AACAGCAGCAGAGACAAATGCACC-3′, 5′- TGCAGTTCCAGAGAGTTCCACACT-3′; ***Acox1***, 5′-GCCCAACTGTGACTTCCATT-3′, 5′-GGCATGTAACCCGTAGCACT-3′; ***MCAD***, 5′-GATCGCAATGGGTGCTTTTGATAGAA-3′, 5′-AGCTGATTGGCAATGTCTCCAGCAAA-3′; ***LCAD***, 5′-CTTGCTTGGCATCAACATCGCAGA-3′, 5′-ATTGGAGTACGCTTGCTCTTCCCA-3′; ***Cpt1a***, 5′-CCTGCATTCCTTCCCATTTG-3′, 5′-TGCCCATGTCCTTGTAATGTG-3′; ***GNMT***, 5′-GCCTACGTTCCCTGCTACTT-3′, 5′-CCACATCTGCACCCAAATGC-3′; ***GAMT***, 5′-CACGCACCTGCAAATCCTG-3′, 5′-TACCGAAGCCCACTTCCAAGA-3′; ***PEMT***, 5′-GAGAACTCGGAAGCTGAGCA-3′, 5′-CAGCACAAACACGAATCCCC-3′; ***BHMT1***, 5′-ACATCAGGGCGATTGCAGA-3′, 5′-CGGGACATGGAAGGGTTG-3′; ***Cyp4A10***, 5′′-AGAGGTGTTTGACCCTTCCAGGTT-3′, 5′-TTGTTTCCCAATGCAGTTCCTGGC-3′; ***Ehhadh***, 5′-CCAATGCAAAGGCTCGTGTT-3′, 5′-GGTAGAAGCTGCGTTCCTCTTG-3′; ***Actb***, 5′-CAGCCTTCCTTCTTGGGTATGG-3′, 5′-CTGTGTTGGCATAGAGGTCTTTACG-3′; ***36B4***, 5′-GGCCCTGCACTCTCGCTTTC-3′, 5′-TGCCAGGACGCGCTTGT-3′. Target gene mRNA levels were quantified and expressed after normalising to either Actb or 36B4 levels.

### Western blot analysis

Portions of liver were excised and snap frozen. Liver tissues were lysed and sonicated in solubilization buffer. Immunoblot analysis was performed as previously described^49^, with some modifications. Blots were incubated with specific antibodies against NNMT Sirt1 (both from Abcam), tubulin (from Millipore), and β-actin (from Sigma).

### Bisulfite Sequencing

Total liver genomic DNA samples were extracted using the GenElute Mammalian Genomic DNA Miniprep Kit (Sigma-Aldrich). Thereafter, samples were bisulfite-modified using the EpiTect Bisulfite Kit (Qiagen, Hilden, Germany). For bisulfite sequencing analysis, bisulfite-modified DNA samples were amplified using primers designed for the regions of CPG island of CTGF, Col4A1 and Col4A2. The primer sequences were: CTGF, 5′-GTAGGTTTTATTAGTTT-3′, 5′-CAAAAAAAACCCTTATATAAATC-3′; Col4A1, 5′-GGGAGGAGTTGGGTG-3′, 5′-AAAACAACAAAAACCAAAC-3′; Col4A2, 5′-GTTTTTAAGGGGAGGATTTTG-3′, 5′-TCACCTCAAAATCAAAAACTACTACC-3′.

PCR products were cloned into pT7Blue T-vector and positive clones were sequenced following the normal protocol of ABI 3130xl sequencer. We used an online quantification tool for methylation analysis (QUMA) (http://quma.cdb.riken.jp/)^50^.

### Determination of SAM and SAH levels

The levels of SAM and SAH, in the livers of experimental animals, were measured using a liquid chromatography (LC)-mass spectrometry (MS)-based technique (A-KIT, Gifu, Japan). The liver of each mouse was homogenized, and the extracts were applied to LC separations by using a Intrada™ Amino Acid, 100 mm × 3 mm column (Imtakt, Kyoto, Japan). Column temperature and flow rate were set to 35 °C and 0.6 mL/min, respectively. The mobile phases were composed of 50% solvent A (90/10 acetonitrile/water containing 0.1% formic acid) and 50% solvent B (10/90 methanol/water containing 100 mM 0.1% ammonium formate). An LCMS-8040 tandem quadrupole mass spectrometer (Shimadzu, Japan) was operated in a positive mode with an ESI source.

### Measurement of NA metabolites

Three volumes of methanol containing 6% perchloric acid and 4% phosphoric acid were added to minced liver or serum samples, and each mixture was vigorously vortexed. After centrifugation, the supernatants were diluted with water and analysed using LC/MS/MS. A Shimadzu Nexera UHPLC system (Shimadzu, Kyoto, Japan), consisting of an LC-30 AD pump, DGU-20A5R degasser, CTO-20AC column oven, and an SIL-30ACMP autosampler, was used. Separation was performed on a Triart C18 column (3.0 × 150 mm, 5 μm, YMC, Kyoto, Japan) at 50 °C. Mobile phase A was water/formic acid/undecafluorohexanoic acid (1000/0.5/0.5, v/v/v) and mobile phase B was methanol. The chromatographic conditions were: 0–4 min (5–80% B, 0.5 mL/min), 4–4.01 min (80–95% B, 0.5–1.0 mL/min), 4.01–7 min (95% B, 1.0 mL/min), 7–7.01 min (95–5% B, 1.0–0.5 mL/min), and 7.01–13 min (5% B, 0.5 mL/min). Mass spectrometric detection was performed using an API5000 triple quadrupole mass spectrometer (SCIEX, Framingham, MA, USA) with electrospray ionization (ESI) operated in positive ion mode; the ESI-MS/MS parameters were optimized using standard solutions for each analyte. Quantitation was performed by multiple reaction monitoring, with the following transitions: *m/z* 123 → 80 for NAM, *m/z* 124 → 80 for NA, *m/z* 137 → 94 for MNA, *m/z* 139 → 78 for nicotinamide-N-oxide, *m/z* 153 → 110 for 2PY, *m/z* 153 → 136 for 4PY, *m/z* 335 → 123 for NMN, and *m/z* 664 → 136 for NAD^+^.

### Measurement of Sirt3 activity

Sirt3 activity was measured in liver mitochondria using the Mitochondria Isolation Kit for Tissue (abcam) and Cyclex SIRT3 Deacetylase Fluorometric kit Ver.2 (MBL, Nagoya, Japan)^51^. Fluorescence intensity was measured by a Cytation 5 plate reader (Bio Tek) (excitation wavelength, 350 nm; emission wavelength, 450 nm).

### Statistical Analysis

Data are expressed as means ± standard errors of measurement, with p < 0.05 being considered statistically significant. One-way analysis of variance was used to determine significant differences among groups. For the overall analysis of variance, the Tukey-Kramer test for multiple comparisons was used to assess individual group differences.

## Electronic supplementary material

Supplementary Material
