# Supplementary material for: NNMT activation can contribute to the development of fatty liver disease by modulating the NAD+ metabolism
Source: Sci Rep. 2018 Jun 5;8:8637. doi: 10.1038/s41598-018-26882-8 (PMC5988709; doi:10.1038/s41598-018-26882-8)

NNMT activation can contribute to the development of fatty liver disease by modulating the NAD$^+$ metabolism

Motoaki Komatsu[1+], Takeshi Kanda[1+], Hidenori Urai[1], Arata Kurokochi[1], Rina Kitahama[1], Shuhei Shigaki[2], Takashi Ono[2], Hideo Yukioka[2], Kazuhiro Hasegawa[1], Hirobumi Tokuyama[1], Hiroshi Kawabe[3], Shu Wakino[1*], and Hiroshi Itoh[1]

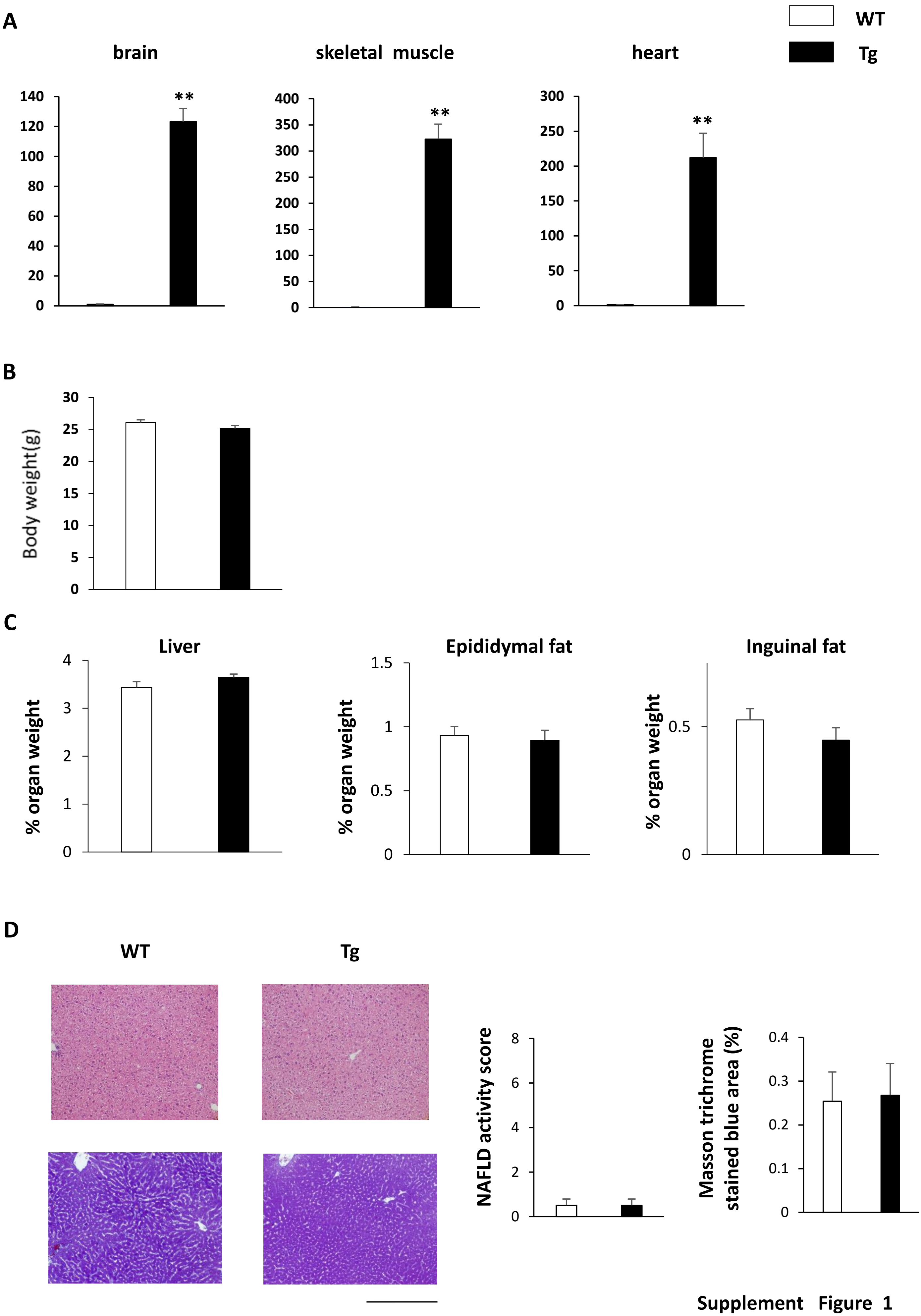

Supplement Figure 1

Supplementary Figure 1. NMMT overexpression does not affect mice on a regular diet.

(A) NNMT mRNA expression levels in brain, skeletal muscle and heart of Tg mice (black bars) and WT littermates (white bars) were measured by real-time PCR (n = 5 per group). (B) Body weight, liver weight/body weight ratio, and epididymal and inguinal fat weight/body weight ratios in NMMT Tg mice (black bars) and WT littermates (white bars) on a regular diet for 3 months (n = 4 per group). (C) H-E staining (upper panel) and Masson trichrome staining (lower panel) of liver from Tg and WT mice on a regular diet for 3 months. Bars represent 1.0 mm. NAFLD activity score and Masson trichrome stained blue area (%) were assessed (n = 4 per group). **P < 0.01 versus WT mice.

Abbreviations: NNMT, nicotinamide N-methyltransferase; Tg, transgenic; WT wild type; Epi, epididymal; Ing, inguinal

Where possible, Supplementary Information (text, tables and images) should be combined and supplied as a single file, preferably in PDF format.

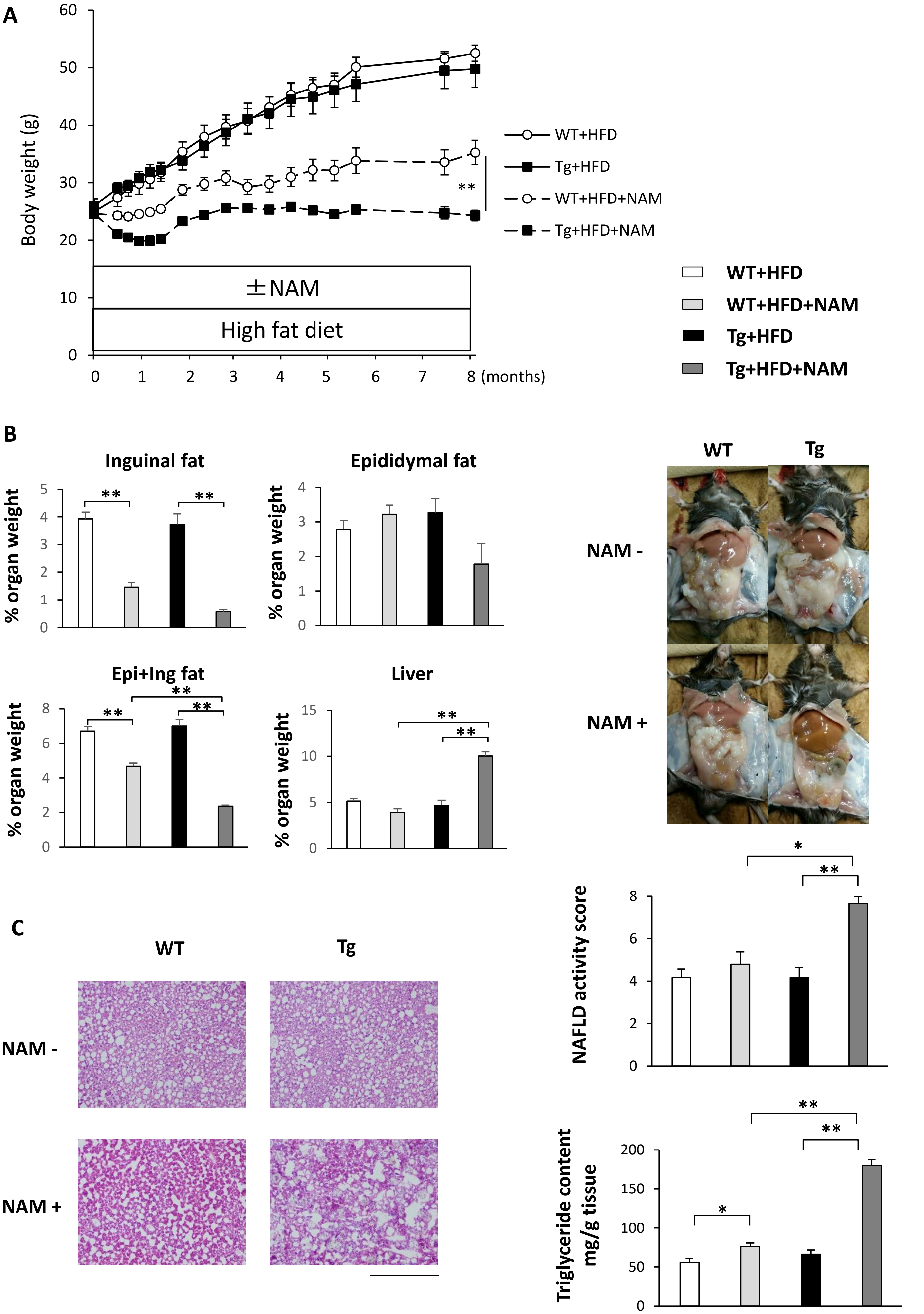

Supplement Figure 2

Supplementary Figure 2. Long-term treatment with NAM induces hepatomegaly in male NNMT Tg mice on a HFD

(A) Body weight after simultaneous administration of an HFD with or without NAM to male NMMT Tg mice and male WT littermates for 8 months (n = 3–8 per group). (B) Ratios of liver, epididymal, inguinal and epi+ing fat weight/body weight in male Tg and WT mice on a HFD with or without NAM (n = 3–8 per group). Mouse abdominal cavity demonstrating hepatomegaly in Tg mice on a HFD with NAM. (C) Representative H-E staining of liver sections from WT and NNMT Tg mice on a HFD with or without NAM. Scale bar represents 100 μm. NAFLD activity score was assessed. Liver triglyceride content of WT and NNMT Tg mice on a HFD with or without NAM for 8 months (n = 3–8 per group). $^{*}P < 0.05$, $^{**}P < 0.01$.

Abbreviations: NNMT, nicotinamide N-methyltransferase; HFD, high fat diet; NAM, nicotinamide; Tg, transgenic; WT wild type; Epi, epididymal; Ing, inguinal

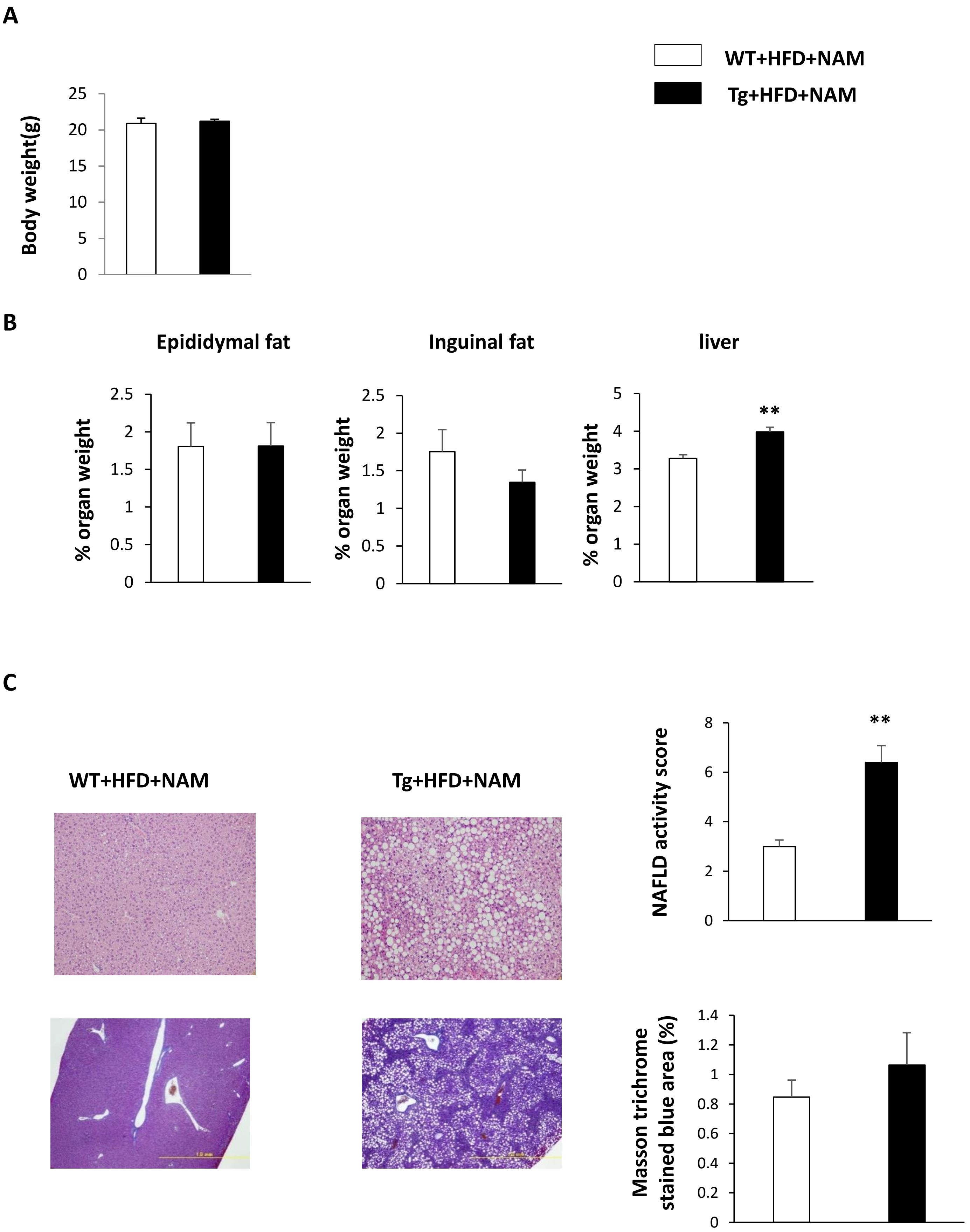

Supplement Figure 3

Supplementary Figure 3. A NAM-supplemented HFD induces hepatomegaly in female NNMT Tg mice.

(A) After 7 weeks of HFD feeding, female Tg mice and WT mice were treated with NAM for 12 weeks (n = 5–8 per group). (B) Ratios of epididymal and inguinal fat weight/body weight in female Tg and WT mice (n = 5–8 per group). The third bar graph shows the liver weight/body weight ratio in female Tg and WT mice (n = 5–8 per group). The black bars represent Tg mice and the white bars represent WT mice on an HFD with NAM. **P < 0.01 versus WT mice. (C) H-E staining (upper panel) and Masson trichrome staining (lower panel) of liver from Tg and WT mice on HFD with NAM. NAFLD activity score and Masson trichrome stained blue area (%) were assessed (n =5-6 per group). **P < 0.01 versus WT mice.

Abbreviations: NNMT, nicotinamide N-methyltransferase; HFD, high fat diet; NAM, nicotinamide; Tg, transgenic; WT wild type; Epi, epididymal; Ing, inguinal

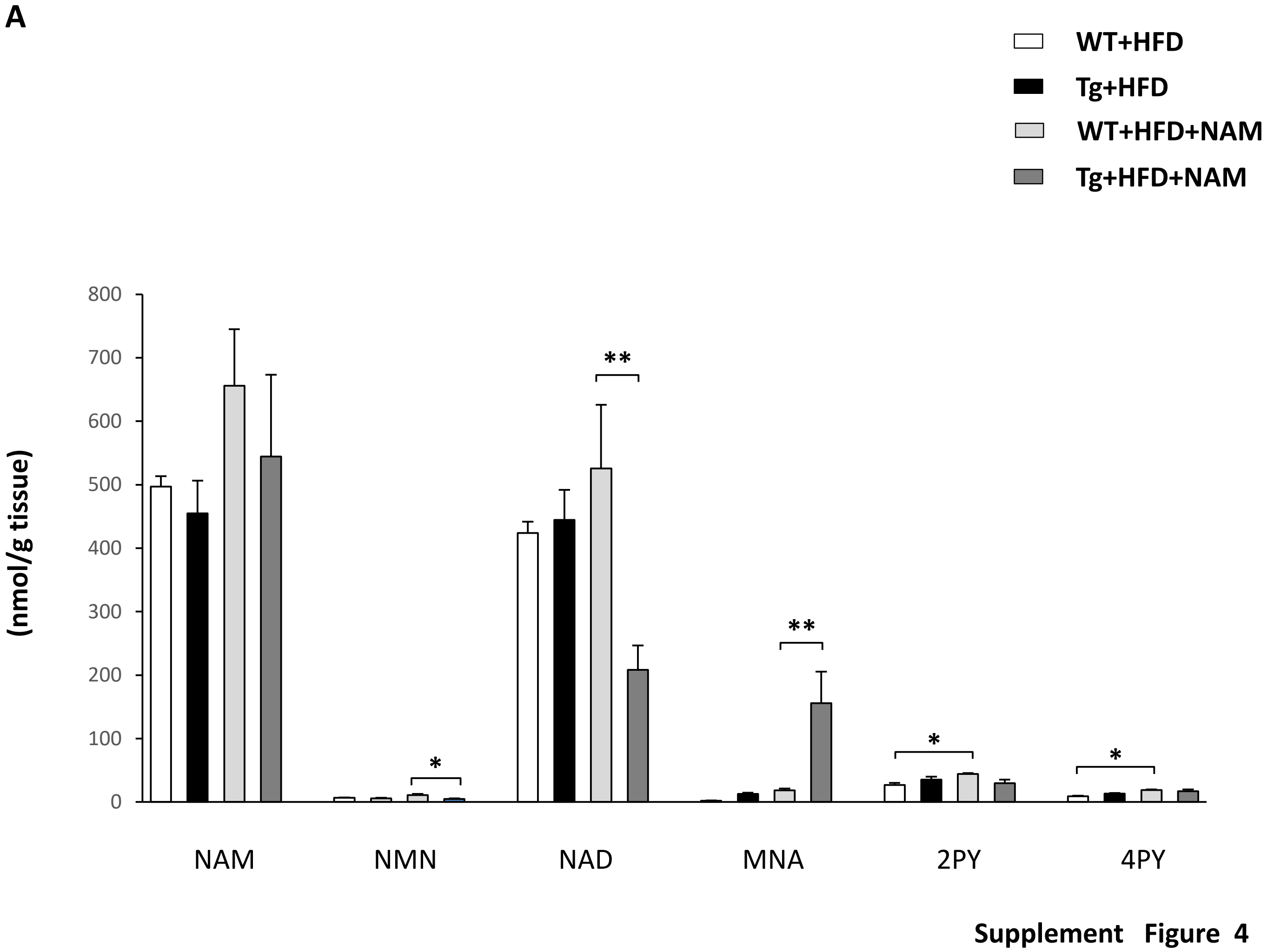

Supplementary Figure 4. Long-term treatment with NAM alters NAD^{+} metabolites

(A) Nicotinic acid metabolites in the livers of NMMT Tg mice and WT littermates on a HFD with or without NAM for 8 months (n = 5–6 per group). The data represent the means ± SEM *P < 0.05, **P < 0.01.

Abbreviations: NAM, nicotinamide; MNA, 1-methylnicotinamide; NNO, nicotinamide-N-oxide; 2PY, N-methyl-2-pyridone-5-carboxamide; 4PY, N-methyl-4-pyridone-5-carboxamide; NMN, nicotinamide mononucleotide; NAD+, nicotinamide adenine dinucleotide; HFD, high fat diet; Tg, transgenic; WT, wild type.

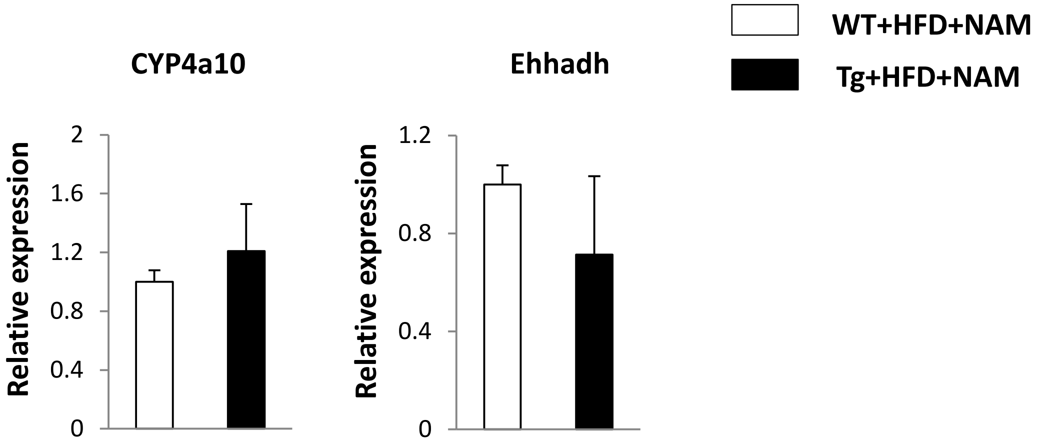

Supplement Figure 5

Supplementary Figure 5. NMMT overexpression does not affect PPARα target genes.
Relative mRNA expression levels in the livers of Tg mice (black bars) and WT mice (white bars) on an HFD with NAM. (n = 5–6 per group). *P < 0.05, **P < 0.01 versus WT mice.

Abbreviations: NNMT, nicotinamide N-methyltransferase; HFD, high fat diet; NAM, nicotinamide; Tg, transgenic; WT wild type; CYP, cytochrome P450; Ehhadh, enoyl-coenzyme a hydratase and 3-hydroxyacyl coenzyme a dehydrogenase.

Supplementary Figure 6. Full-length blots/gels are presented below

Figure 2B

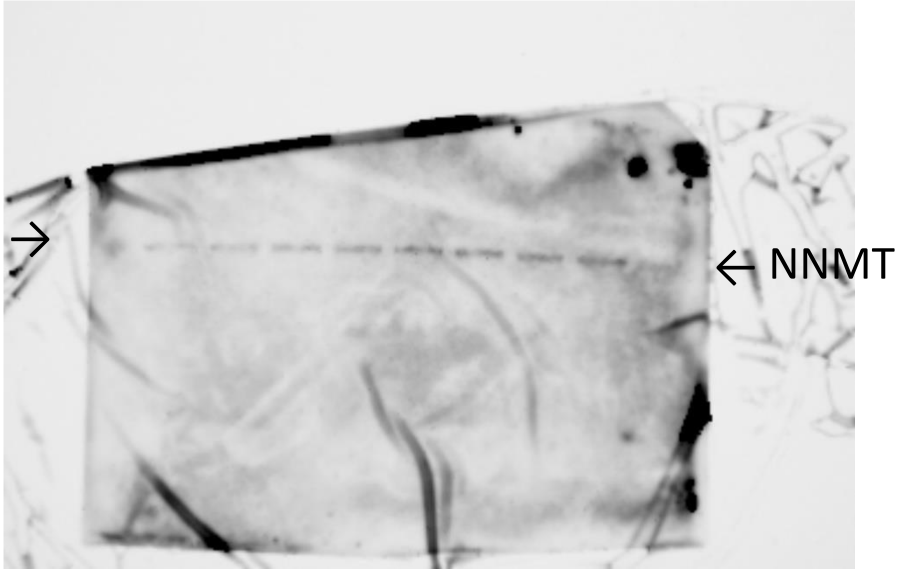

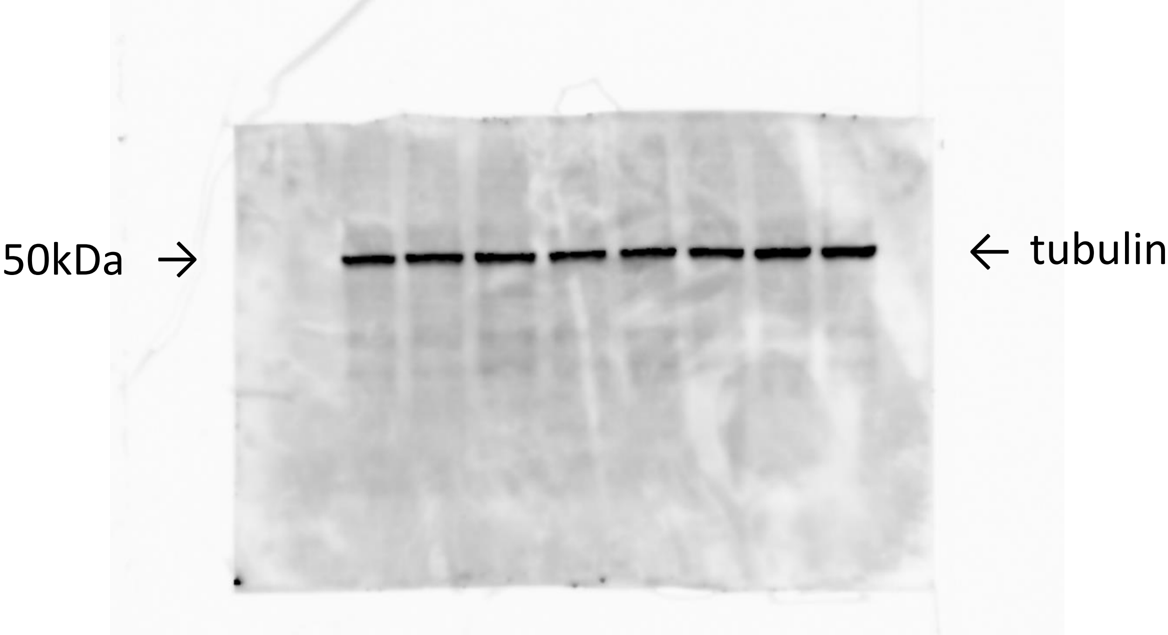

Figure 2C

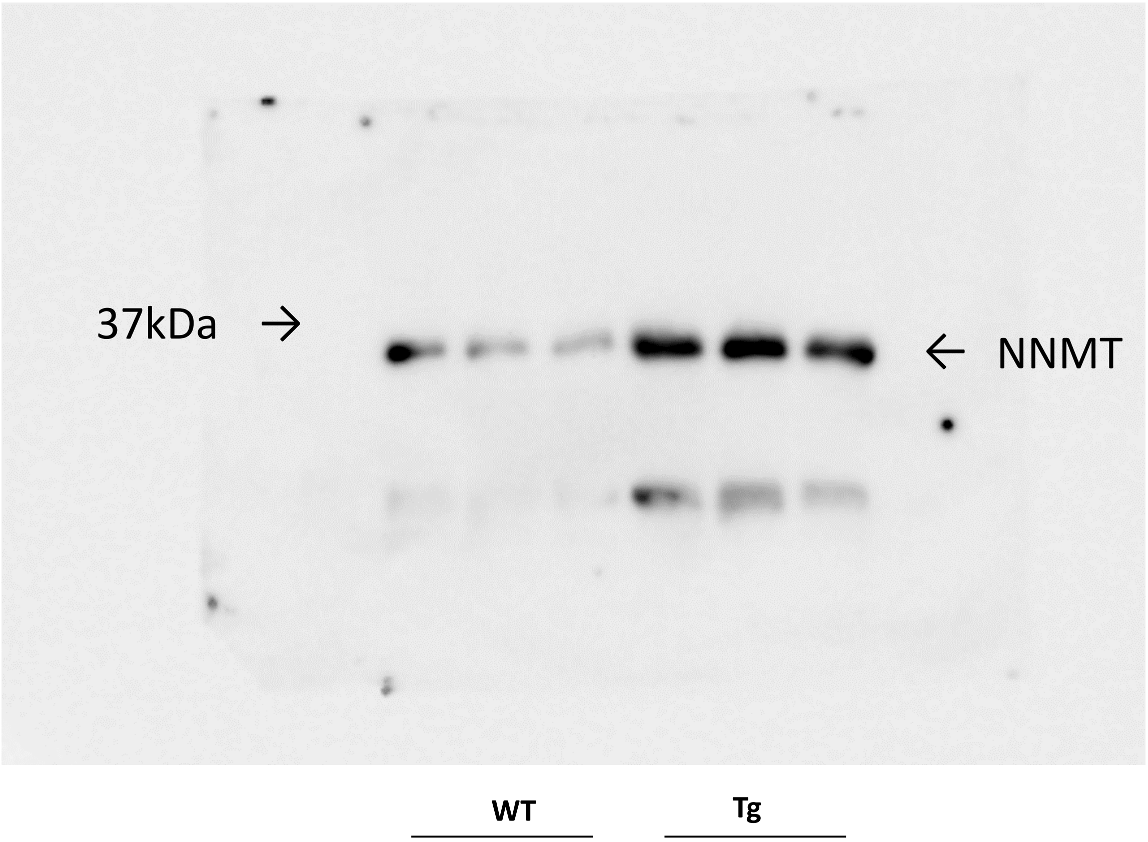

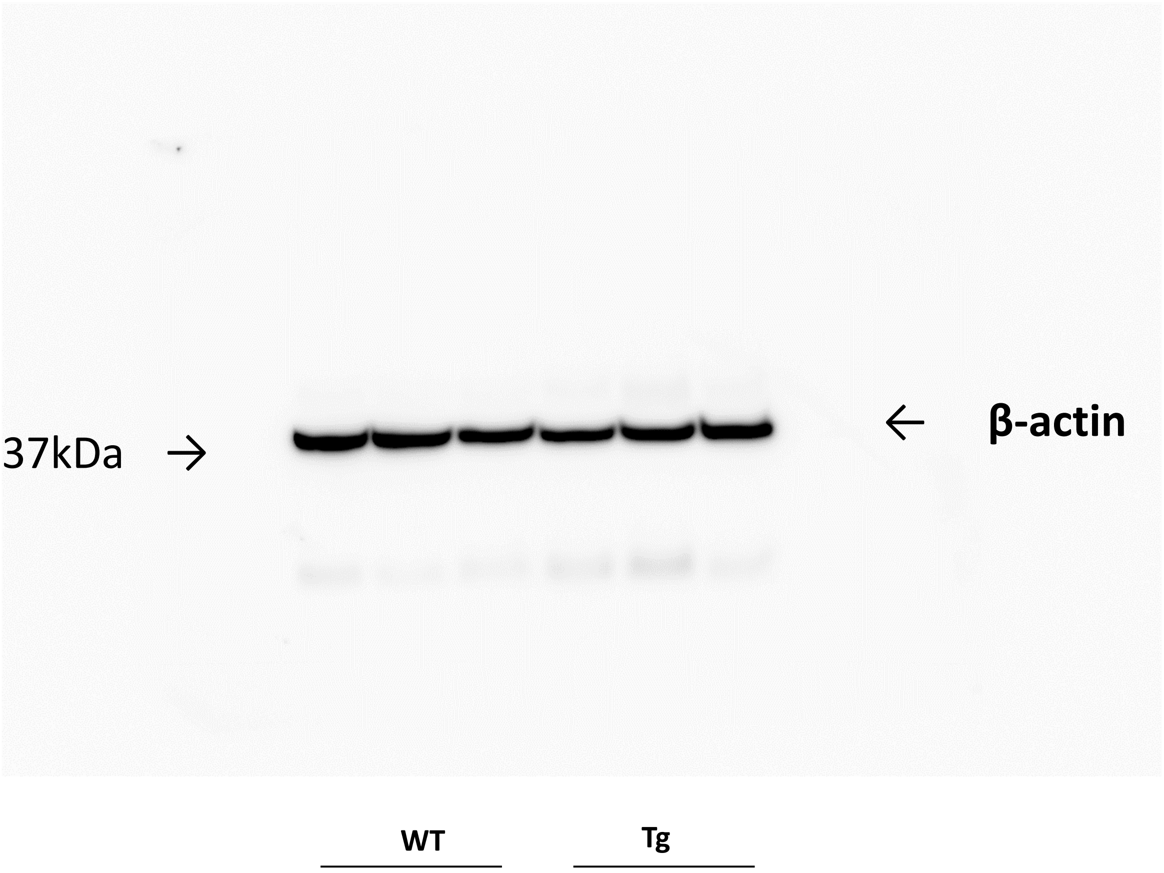

Figure 6C

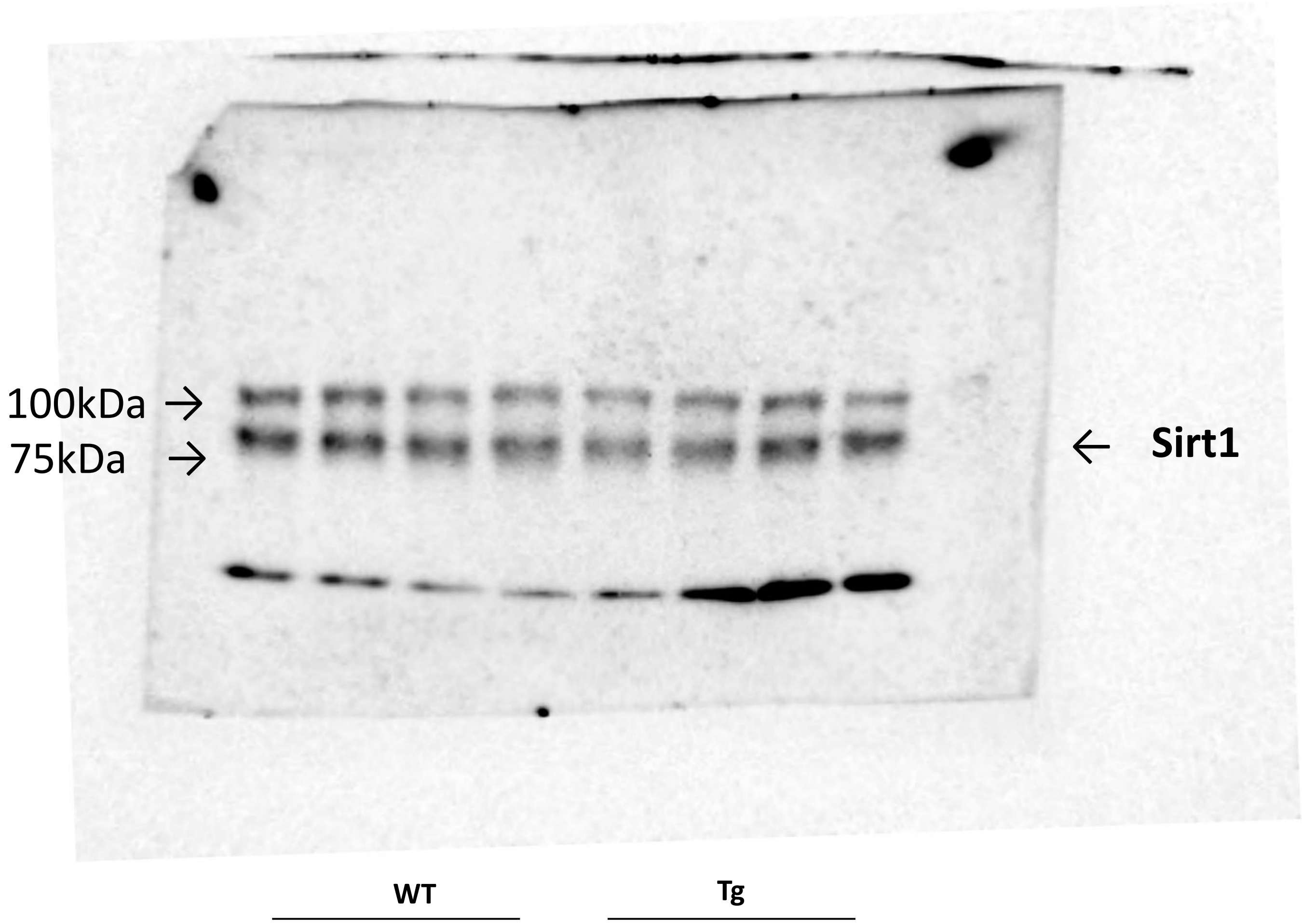

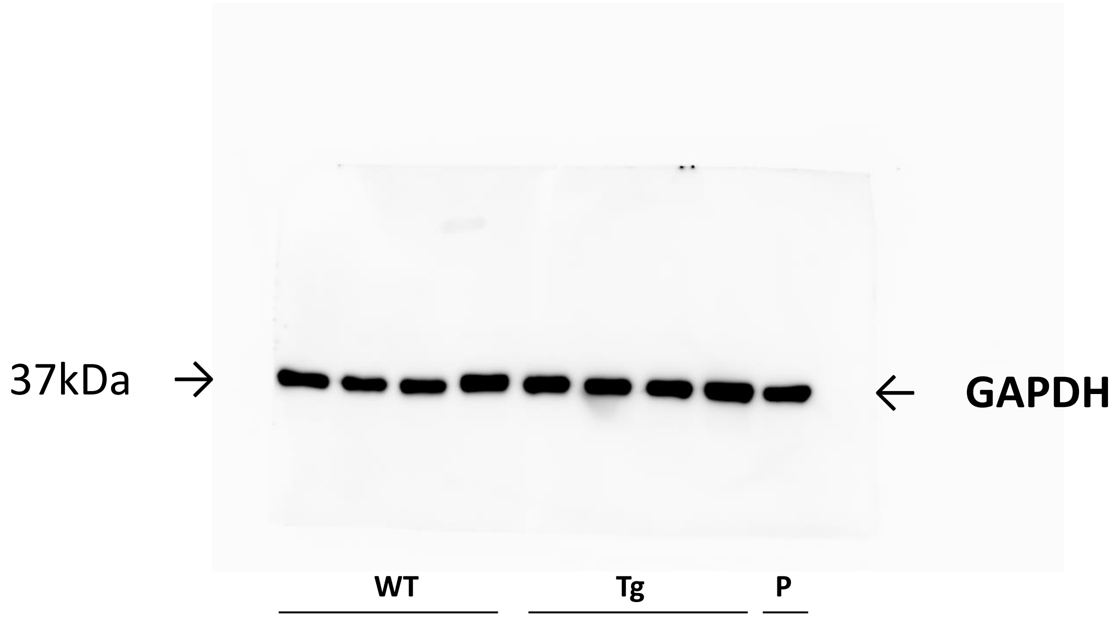

Supplement: Supplementary file 1 — Supplementary Material [file 41598_2018_26882_MOESM1_ESM.pdf]
